# Immune correlates of postexposure vaccine protection against Marburg virus

**DOI:** 10.1038/s41598-020-59976-3

**Published:** 2020-02-20

**Authors:** Courtney Woolsey, Allen Jankeel, Demetrius Matassov, Joan B. Geisbert, Krystle N. Agans, Viktoriya Borisevich, Robert W. Cross, Daniel J. Deer, Karla A. Fenton, Theresa E. Latham, Cheryl S. Gerardi, Chad E. Mire, John H. Eldridge, Ilhem Messaoudi, Thomas W. Geisbert

**Affiliations:** 1 Department of Microbiology and Immunology, University of Texas Medical Branch, Galveston, TX 77555 USA; 2 Galveston National Laboratory, University of Texas Medical Branch, Galveston, TX 77555 USA; 3 Department of Molecular Biology and Biochemistry, School of Biological Sciences, University of California, Irvine, Irvine, CA 92697 USA; 4 Department of Virology and Vaccine Vectors, Profectus BioSciences Inc., Pearl River, NY 10965 USA; 5 Department of Immunology, Profectus BioSciences Inc., Pearl River, NY 10965 USA

**Keywords:** Live attenuated vaccines, Marburg virus

## Abstract

Postexposure immunization can prevent disease and reduce transmission following pathogen exposure. The rapid immunostimulatory properties of recombinant vesicular stomatitis virus (rVSV)-based vaccines make them suitable postexposure treatments against the filoviruses Ebola virus and Marburg virus (MARV); however, the mechanisms that drive this protection are undefined. Previously, we reported 60–75% survival of rhesus macaques treated with rVSV vectors expressing MARV glycoprotein (GP) 20–30 minutes after a low dose exposure to the most pathogenic variant of MARV, Angola. Survival in this model was linked to production of GP-specific antibodies and lower viral load. To confirm these results and potentially identify novel correlates of postexposure protection, we performed a similar experiment, but analyzed plasma cytokine levels, frequencies of immune cell subsets, and the transcriptional response to infection in peripheral blood. In surviving macaques (80–89%), we observed induction of genes mapping to antiviral and interferon-related pathways early after treatment and a higher percentage of T helper 1 (Th1) and NK cells. In contrast, the response of non-surviving macaques was characterized by hypercytokinemia; a T helper 2 signature; recruitment of low HLA-DR expressing monocytes and regulatory T-cells; and transcription of immune checkpoint (e.g., *PD-1*, *LAG3*) genes. These results suggest dysregulated immunoregulation is associated with poor prognosis, whereas early innate signaling and Th1-skewed immunity are important for survival.

## Introduction

Members of the genera *Marburgvirus* (MARV) and *Ebolavirus* (EBOV) are pathogens in the family *Filoviridae* that cause a similar life-threatening hemorrhagic disease in humans and non-human primates (NHPs)^1^. More than 30,000 people have been infected with EBOV, whereas 469 cumulative cases and 376 recorded deaths are attributed to Marburg virus disease (MVD)^2–4^. Although fewer cases are recorded for MARV, future outbreaks and spread of the virus into non-endemic regions are of great concern. MVD has an overall mortality rate of 81% and imported cases have occurred in Germany, the former Yugoslavia (presently Serbia), the Netherlands, and the United States^1–4^. Moreover, the Egyptian fruit bat host reservoir has a wide geographic distribution^5^. While MARV is thought to be limited to equatorial Africa, a research group that surveyed a large South African bat colony found that ~53% of these animals were seropositive for the virus, and recently MARV was isolated from bats in West Africa for the first time^6,7^. Surveillance in the latter region also revealed serological evidence of filoviruses (MARV and EBOV) circulating in human subjects prior to the 2013–2016 EBOV outbreak^8,9^. The likelihood of spillover events and spread into human populations emphasizes the need for adequate countermeasures against this deadly virus.

One of the most promising vaccine candidates against MARV and EBOV uses a live, attenuated recombinant vesicular stomatitis virus (rVSV) platform to express filovirus glycoprotein (GP) antigen. Results from human clinical trials for an EBOV GP-based rVSV manufactured by Merck showed favorable safety and immunogenicity profiles. Administration of this vaccine to contacts and contacts of contacts in a cluster-randomized ring vaccination trial during the West African outbreak prevented disease in 100% of those immunized within 10 days onwards, emphasizing the utility of rVSV vectors for emergency interventions^10^. Moreover, preliminary results from the ring vaccination trial for the ongoing Ebola outbreak in the Democratic Republic of Congo indicate this vaccine is 97.5% effective for those with onset of illness 10 day or more post-immunization and 88.1% effective overall for the >93,965 people that have been vaccinated^11^. A similar strategy could be implemented to prevent disease and reduce community transmission in the event of a MARV outbreak.

Efficacy studies for rVSV vaccines against MVD have largely been conducted in non-human primates (NHPs), which most accurately recapitulate human infection. A single intramuscular (i.m.) injection of an rVSV expressing the Musoke variant GP (rVSV∆G/MARV-Musoke-GP; ~5e^7^ plaque-forming units (PFU)) or Angola variant GP (rVSV∆G/MARV-Angola-GP; ~5e^7^ PFU) was 100% effective in cynomolgus macaques against a 1000 PFU uniformly lethal MARV challenge when administered within 28 days before challenge^12,13^. A ~2e^7^ PFU dose of the rVSV∆G/MARV-Musoke-GP vaccine also provided cross-protection against the Angola variant and related Ravn virus at the same challenge dose^14^. Furthermore, rVSV∆G/MARV-Musoke-GP (~1–2e^7^ PFU) protected 100% of rhesus macaques when administered 20–30 minutes postexposure following a homologous 1000 PFU MARV-Musoke challenge^12^. If the initial treatment time was extended to 24 and 48 hours after exposure, 83% and 33% survived, respectively^15,16^. Unfortunately, treatment with rVSV vectors expressing MARV-Angola-GP failed to adequately defend macaques against a high dose of the most virulent variant Angola when administered 20–30 minutes after infection. Only 25% of NHPs survived a high 1000 PFU challenge, whereas 60–75% survived a low 50 PFU challenge^17^. Treated survivors had fewer clinical signs of disease, reduced viremia, and high titers of anti-MARV GP IgG, whereas treated NHPs that succumbed failed to generate a vaccine-mediated humoral response.

Understanding the mechanisms that contribute to treatment protection or failure can be useful in determining the appropriate immunomodulatory approaches that can be implemented to enhance postexposure immunity and also to inform rational design of next-generation vaccines. Few studies have examined correlates of rVSV-mediated immunity against filovirus disease, particularly in the postexposure context. While total antibody level against the GP is a reliable predictor of protection, neutralizing ability does not appear to be a necessity^12,14,16–20^. In contrast, cellular responses are not considered critical according to the limited descriptions that exist^12–14,16,18–20^. Early investigations of the rVSV-based immune mechanisms of protection against EBOV have primarily been examined via antibody-mediated immune cell depletion. These studies revealed that CD4+ T-cell depletion during vaccination, unlike CD4+ depletion during challenge, rescinded production of GP-specific IgG and protection against a subsequent EBOV challenge^21^. These results suggest higher participation of CD4+ T-cells in helper functions, such as B-cell maturation and antibody isotype class switching, rather than effector functions. CD8+ T-cells were dispensable against EBOV during rVSV vaccination indicating cellular responses are less essential mediators of rVSV-induced immunity. However, these reports are based on protection following vaccination 28 days before challenge, not postexposure.

Paradoxically, transcriptomic analyses revealed a previously unrecognized role of T-cell immunity in rVSV vaccine protection against EBOV and MARV in the macaque model^13,22^. Likewise, results from clinical trials show rVSV immunization elicited GP-specific T helper cells and cytotoxic T-cell lymphocytes (CTLs) in human subjects^23^. IFN-gamma was the most abundant analyte secreted by these cells suggesting a predominant T helper 1 (Th1)-associated response. Another study showed that survivors during the West African Ebola epidemic had higher T-cell receptor diversity^24^. Effector T-cells, e.g. Th1 cells, might thus have a more meaningful function in combatting infection than presently realized. More experiments are needed given the results of these studies are seemingly contradictory. Protection is probably multifactorial, including elements such as inherent differences in host immunity, vaccine dose or choice, challenge inoculum or the virus species/variant, and the time of vaccination or treatment. Immune mediators might also differ for postexposure treatment compared to prophylactic vaccine protection as the host may adapt to overcome certain cell deficits if a memory response is pre-formed.

As macaques treated with rVSV vectors expressing MARV-Angola-GP are incompletely protected against a low dose challenge of the MARV-Angola variant, we used this experimental model to aid in the identification of specific cell populations and transcriptional correlates that support defense against MVD. Samples from survivors versus subjects that succumbed were compared using RNAseq, flow cytometry, and cytokine bead arrays. The magnitude and quality of the humoral response were measured with MARV GP-specific IgM and IgG enzyme-linked immunosorbent assays (ELISAs) and plaque reduction neutralization tests (PRNT_50_). These analyses add to the limited information pertaining to the systemic host response following MARV infection and rVSV postexposure vaccination. We hypothesize survival is dependent on early induction of innate and adaptive immunity.

## Results

### rVSV treatment affords postexposure protection against a low dose MARV-Angola challenge

To corroborate our previous results demonstrating rVSV as an effective postexposure prophylactic, rhesus macaques (N = 18) were subjected to a low but lethal 50 PFU target dose of MARV-Angola and treated with one of two vectors 20–30 minutes later. Three control animals were left untreated and a single animal received an rVSV expressing HIV gag gene, rVSVN4CT1-HIVgag to account for irrelevant effects of the vector. Treated cohorts received a 10 million PFU i.m. injection of either rVSV∆G/MARV-Angola-GP (henceforth referred to as ∆G; N = 9) or rVSVN2CT1-MARV-Angola-GP (N2; N = 5). The ∆G vector expresses a MARV-Angola-GP from the site of the native VSV glycoprotein (G) gene, whereas the N2 vector expresses the MARV-Angola-GP from the first open reading frame. Unlike ∆G, N2 contains an attenuated G with a truncated cytoplasmic tail. Figure S1a illustrates the design of each vector. Survival data, viremia, clinical findings, and antibody titers for the untreated controls (N = 3), vector control (N = 1) and a cohort of ∆G-treated subjects (N = 4) were published previously (Fig. S1b and Table 1)^17^. Control macaques were shared among studies based on Institutional Animal Care and Use Committee (IACUC) recommendations and ethical concerns. However, two of the historical untreated controls were challenged with an identical virus stock at the same time as the remaining ∆G (N = 5) animals, and the vector control was challenged simultaneously with the N2-treated (N = 5) animals. Therefore, at least one control was challenged at the same time as each new treated cohort to validate the lethality of the challenge material. Back titration titers ranged from 45–80 PFU for the MARV inoculum indicating challenge doses were comparable among the studies.

**Table 1:** Clinical findings.

Animal # Weight Sex	Clinical Observations	GP-specific IgM	GP-specific IgG	Final Outcome	Reference
Untreated Control 1* 5.04 kg Female	Fever (6), depression (6–8), anorexia (7,8), lymphopenia (3,6), ALT +++ (6), AST +++ (6) > (8), ALP + (6), GGT + (6), CRP increase (3,6), tPA +++ (6), PAI-1 +++ (6)	None	None	Succumbed 8 DPI	^17^
Untreated Control 2* 4.04 kg Female	Fever (6), depression (8,9), anorexia (7–9), mild to moderate petechial rash (8,9), BUN ++ (9), CRE + (9), ALT ++ (6) +++ (9), AST +++ (6,9), ALP ++ (9), GGT +++ (9), CRP increase (9), p-selectin +++ (9), d-dimer + (9), tPA + (6) +++ (9), PAI-1 + (6) +++ (9), factor IX + (3)	None	None	Succumbed 9 DPI	^17^
Untreated Control 3* 4.54 kg Female	Fever (6), depression (10), anorexia (8,10), mild to moderate petechial rash (10), emesis (8), leukocytosis (6,10), granulocytosis (3,6), CRE + (10), ALT +++ (10), AST + (6) +++ (10), ALP +++ (10), GGT +++ (10), CRP increase (6,10), d-dimer ++ (6), tPA + (3) ++ (6), PAI-1 ++ (6,10)	None	None	Succumbed 10 DPI	^17^
Vector Control* 4.76 kg Male	Depression (12), anorexia (10–12), mild petechial rash (10–12), BUN + (12), CRE ++ (12), ALT +++ (10,12), AST +++ (10,12), ALP ++ (10,12), GGT +++ (10,12), CRP increase (10,12), d-dimer +++ (3,6,10,12), tPA ++ (10) +++ (12), sCD40L + (10), PAI-1 + (10) +++ (12), factor IX ++ (3)	None	None	Succumbed 12 DPI	^17^
∆G Treated Fatal* 5.56 kg Female	Fever (6), depression (10,11), anorexia (8,9,11), mild to moderate petechial rash (10), ecchymotic rash (11), leukocytosis (11), lymphopenia (6,10), granulocytosis (3,6,10,11), thrombocytopenia (10), BUN + 10) +++ (11), CRE +++ (11), ALT > (10,11), AST > (10,11), ALP ++ (10,11), GGT +++ (10,11), CRP increase (6,10,11), p-selectin + (6) ++ (3) +++ (10,11), d-dimer + (11), PSGL-1 + (3,10), tPA ++ (10) +++ (11), PAI- 1 +++ (10,11), factor IX ++ (10)	None	None	Succumbed 11 DPI	^17^
∆G Survivor 1* 3.64 kg Male	Granulocytosis (21), p-selectin + (3,10) ++ (6), PSGL-1 + (3,6,14,21,28), factor IX + (14)	100 (10), 200 (14), 100 (21), 100 (28)	800 (10), 3,200 (14), 3,200 (21), 6,400 (28)	Survived	^17^
∆G Survivor 2* 3.70 kg Male	CRP increase (28), p-selectin + (14) ++ (28), PSGL-1 + (6) ++ (21,28)	200 (10), 200 (14), 100 (21), 100 (28)	800 (10), 800 (14), 1600 (21), 6400 (28)	Survived	^17^
∆G Survivor 3* 4.80 kg Female	PSGL-1 + (6,10), factor IX + (6,14,21,28) ++ (10)	100 (10), 100 (14), 100 (21)	800 (10), 1,600 (14), 3,200 (21), 6,400 (28)	Survived	^17^
∆G Survivor 4 4.06 kg Female	None	100 (10), 100 (14)	3,200 (10), 3,200 (10), 6,400 (21), 6,400 (28)	Survived	
∆G Survivor 5 4.30 kg Female	Fever (6), mild depression (8,9), mild to moderate petechial rash (8,9,10,11), lymphopenia (6), ALT + (21) +++ (10,14), AST + (14) +++ (10), ALP ++ (10), GGT + (14,21) +++ (10), CRP increase (10), tPA ++ (10), PAI-1 ++ (14) +++ (10), factor IX + (3) ++ (21) +++ (6,10,14)	1600 (10), 800 (14), 200 (21), 100 (28)	100 (6), 1,600 (10), 3,200 (14), 6,400 (21), 12,800 (28)	Survived	
∆G Survivor 6 4.06 kg Female	Thrombocytopenia (28), p-selectin + (3) ++ (28), tPA + (28), PAI-1 +++ (28)	100 (10), 200 (14), 100 (21)	400 (10), 1,600 (14), 1,600 (21), 6,400 (28)	Survived	
∆G Survivor 7 4.60 kg Female	Granulocytosis (6), factor IX + (3,14)	100 (10), 100 (14)	100 (6), 1,600 (10), 1,600 (14), 3,200 (21), 3,200 (28)	Survived	
∆G Survivor 8 4.36 kg Female	d-dimer ++ (28), sCD40L ++ (10,14,21) +++ (3,6,28), factor IX + (3,6,21,28)	100 (10), 100 (14)	100 (6), 1,600 (10), 1,600 (14), 1,600 (21), 3,200 (28)	Survived	
N2 Treated Fatal 5.10 kg Male	Depression (12,13,14), anorexia (12), mild to moderate petechial rash (13,14), mild dyspnea (14), lymphopenia (10), granulocytosis (10,14), thrombocytopenia (10,14), BUN ++ (14), CRE + (14), ALT + (10) +++ (14), AST ++ (10) +++ (14), ALP ++ (14), GGT + (14), CRP increase (10,14), p-selectin + (6), d-dimer + (6,10,14), tPA +++ (14), PAI-1 + (3) ++ (10) +++ (14), factor IX + (14)	None	200 (14)	Succumbed 14 DPI	
N2 Survivor 1 4.28 kg Male	Fever (6), CRP increase (6)	100 (6), 400 (10), 400 (14), 200 (21), 100 (28)	1,600 (10), 3,200 (14), 3,200 (21), 3,200 (28)	Survived	
N2 Survivor 2 4.94 kg Female	ALT + (6,10,14)	100 (6), 100 (10), 100 (14)	800 (10), 800 (14), 1,600 (21), 1,600 (28)	Survived	
N2 Survivor 3 4.78 kg Female	P-selectin +++ (10,14), d-dimer + (10,14), tPA +++ (10,14), PAI-1 +++ (10,14)	100 (6), 100 (10), 100 (14)	100 (6), 3,200 (10), 3,200 (14), 6,400 (21), 6,400 (28)	Survived	
N2 Survivor 4 4.22 kg Female	ALT ++ (10), d-dimer + (3,6,14,21,28), PSGL-1 + (3), tPA + (3,14), sCD40L + (14) ++ (3,28), PAI-1 + (3), factor IX + (10) ++ (3,14,28)	200 (10)	800 (10), 1,600 (14), 1,600 (21), 3,200 (28)	Survived	

Animals were monitored daily for clinical signs. Hematological changes, serum biochemistry values, and reciprocal dilution anti-MARV-GP IgM and IgG titers were evaluated in NHP subjects at 0, 3, 6, 10, 14, 21, and terminally, or at 28 days after challenge. The DPI for each parameter is indicated in parentheses. An asterisk (*) denotes a historical sample. Fever: temperature greater than 3.0 °F above baseline or at least 1.5 °F above baseline and ≥104.0 °F. Lymphopenia and thrombocytopenia: ≥35% drop in numbers of lymphocytes, and platelets respectively. Leukocytosis and granulocytosis: ≥ two-fold increase in leukocytes and granulocytes respectively. Crosses indicate increases in liver enzymes (ALT, AST, ALP, GGT), renal function test values (BUN, CRE), or coagulation-associated analytes (p-selectin, d-dimer, PSGL-1, tPA, sCD40L, PAI-1, factor IX): 2- to 3-fold increase, +; >3- up to 5-fold increase, ++; >5 fold increase, +++; out of range, >. CRP increase refers to samples with concentrations >10 mg/L. Abbreviations: ∆G (referring to individual monkey treated with rVSV∆G/MARV-Angola-GP); N2 (referring to individual monkey treated with rVSVN2CT1-MARV-Angola GP); BUN (blood urea nitrogen); CRE (creatinine); ALT (alanine aminotransferase); AST (aspartate aminotransferase); ALP (alkaline phosphatase); GGT (gamma-glutamyltransferase); CRP (c-reactive protein); PSGL-1 (p-selectin glycoprotein ligand-1); tPA (tissue plasminogen activator); sCD40L (soluble CD40-ligand); PAI-1 (plasminogen activator inhibitor-1); IgM (immunoglobulin M); IgG (immunoglobulin G); DPI (days post-infection).

MARV-infected animals were monitored daily for signs of illness up to the 28 days post-infection (DPI) study endpoint. To assess viremia and disease state, temporal blood samples were taken pre-challenge, 3, 6, 10, 14, 21, and 28 post challenge, or terminally. The three untreated controls succumbed 8–10 DPI, whereas the vector control was euthanized on 12 DPI (Fig. S1b). Survival of groups treated with rVSV vectors expressing MARV-Angola-GP was significantly different than the untreated control group with a survival rate of 89% (log-rank test p ≤ 0.001) for ∆G-treated macaques (N = 9) and 80% (p ≤ 0.01) for those receiving N2 treatment (N = 5). No statistical difference was found between the ∆G and N2 groups (p = 0.7053). The median time-to-death was 9 DPI for the untreated controls and 12 DPI for the vector control and treated fatal subjects, respectively (p < 0.05). This suggests rVSV treatment delayed disease progression or the onset of illness.

As expected, survival was associated with fewer clinical indications of disease such as fever, depression, anorexia, and hematological changes (Table 1). Elevated liver-associated enzymes (alanine aminotransferase (ALT), aspartate aminotransferase (AST), alkaline phosphatase (ALP), gamma-glutamyltransferase (GGT)) and kidney-associated products (blood urea nitrogen (BUN) and creatinine (CRE)) were prominent in the serum of fatal cases indicating potential organ damage. C-reactive protein (CRP) values were also increased in these animals signifying inflammation. Other than Control 1, all non-surviving animals exhibited hemorrhagic manifestations of disease including the formation of a petechial rash and/or thrombocytopenia. Fatal animals also exhibited higher plasma levels of coagulation-associated products including d-dimers and plasminogen activator inhibitor-1 (PAI-1) (Fig. S2). Of the twelve treated animals that did not succumb, only one displayed considerable signs that were consistent with MVD: ∆G Survivor 5. The other survivors remained relatively healthy other than a transient fever in one animal and a temporary rise of ALT in two N2-treated subjects. Many treated survivors also exhibited some coagulation abnormalities. No statistically significant difference was associated with sex (Fisher’s exact test; p > 0.9999) or weight (Mann-Whitney test; p = 0.0794) for the animals when comparing outcome (survival).

### rVSV postexposure defense is dependent on antibody production

Humoral responses were assessed by ELISA and PRNT_50_ assays. In line with past reports, only treated survivors formed anti-MARV-GP IgM and IgG antibodies with both immunoglobulin classes appearing within 6–10 DPI (Table 1)^15,17,18^. Low IgM titers (1:100 to 1:1,600) generally declined during the convalescent stage (21 and 28 DPI) conjointly with increasing moderate to high titers of IgG (1:1,600 to 1:12,800). Treated survivors produced low levels of neutralizing antibody (Table S1) with PRNT_50_ values ranging from 1:20 to 1:40. Neutralizing antibody for many survivors did not appear until after 10 DPI indicating direct virus neutralization may not be an immediate requirement for protection.

### rVSV-treated animals have a lower viral load

**Figure 1:**
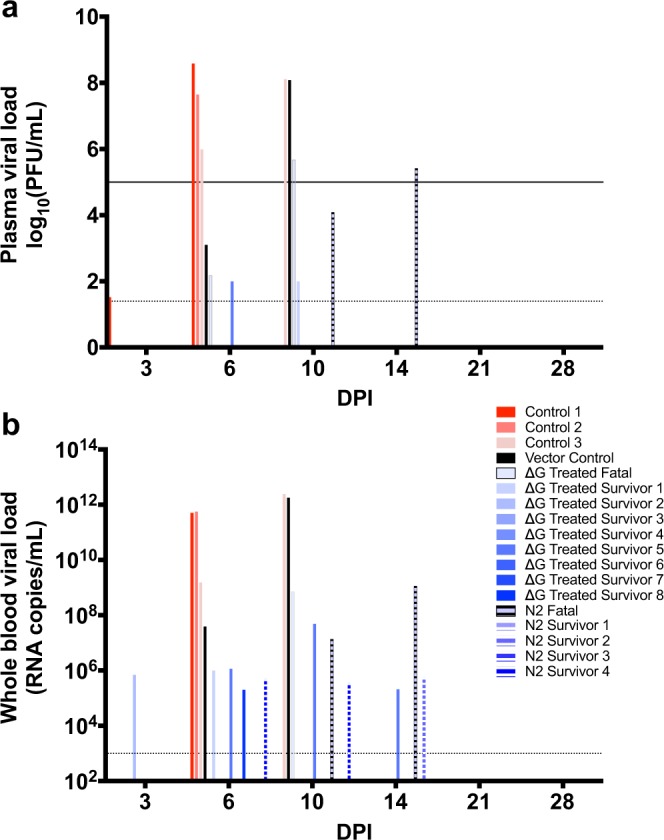
MARV viral loads. (**a**) A standard plaque assay was used to determine infectious titers in the plasma of MARV-Angola infected animals on 3, 6, 10, 14, 21, and/or 28 DPI. The solid black line denotes the viremia threshold that dictates survival. (**b)** Whole blood titers were determined via RT-qPCR. Shown are individual untreated (red-pink bars), rVSV∆G/MARV-Angola-GP-treated (∆G; solid blue gradient bars), and rVSVN2CT1-MARV-Angola-GP-treated (N2; horizontal stripe blue gradient bars) subjects, as well as the single vector control (black bar). The limit of detection is 25 PFU/mL for the plaque assay and 1000 copies/mL for RT-qPCR (dotted line). Abbreviation: PFU (plaque-forming units); DPI (days post-infection).

Similar to our prior results^15–18^, treated survivors had decreased viral loads compared to animals that succumbed (two-tailed t-test p < 0.0001 at peak viremia) (Fig. 1a). Low plasma viremia ( < 2 log10 pfu/mL) was detected in two of the twelve survivors: ∆G Survivors 1 and 5 at 10 and 6 DPI, respectively. Comparatively, ~6 to 8 log10 pfu/mL of infectious MARV was observed in plasma of the untreated control group at 6 DPI. Viral titers were 4–7 logs less for the treated non-survivors and ~3–5 logs lower for the vector control at this time point. By 10 DPI, the vector and remaining untreated control reached ~ 8 logs, whereas treated animals that did not survive ranged from ~4 to 6 logs. Based on these data, the threshold of circulating infectious virus that dictates therapeutic vaccine-induced protection in this model is approximately 5 logs.

A similar pattern was observed for RT-qPCR (Fig. 1b). Untreated controls and the vector control reached 11–12 logs of viral RNA copies/mL in whole blood at end-stage disease. In contrast, terminal titers in treated fatal cases were ~1 to 3 logs less. MARV RNA was present in the blood of approximately half of the treated survivors but never exceeded 7 logs. The untreated controls, vector control, and treated fatal animals reached high titers of ~8–11 logs in all tissues tested (Table S2). Despite no infectious virus or antigen detection at the study endpoint, viral copies were detected in most of the major organs for all of the treated survivors suggesting delayed clearance of viral genomic components in these tissues. PCR titers ranged from ~5–7 log viral RNA copies/mL and were reduced compared to non-surviving subjects in adrenal gland, liver, and spleen tissues (multiple two-tailed t-tests p < 0.05).

### Normalization of samples

Viral load is a strong predictor of filovirus disease progression and lethality in humans and NHP^25–28^. Thus, this parameter was used to normalize samples into early, mid, and late disease stages for RNAseq, flow cytometry, and our cytokine bead array analyses (Table S3). Normalization was implemented on account of the inconsistency in disease onset in subjects, which could potentially confound an analysis strategy solely based on DPI. Treatment administration or host factors (e.g. age, sex, variable outbred response) may contribute to these variances. Our normalization strategy is line with other transcriptomic studies in macaques that demonstrated the presence of three discernible stages of MARV infection, with mid-disease corresponding to first detection of viremia and clinical signs and late-disease corresponding to peak viremia at 0–2 days before death^27,28^. In this study, the median time points for mid- and late-disease in fatal cases were 6 and 10 DPI, respectively (Table 1).

Since the goal of the study was to identify shared survivor or fatal signatures and not treatment-specific differences, we combined samples into a “survivor” or “fatal” dataset to compare host immune responses. The survivor group (N = 12) included all animals treated with ∆G and N2 vectors that survived, as there was no statistical difference between these groups in terms of survival. The untreated controls, the vector control, and rVSV-treated non-survivors were combined into a single fatal dataset (N = 6). One limitation of this study is that we were unable to directly compare vaccinated survivors and non-survivors due to the limited number of the latter. However, no major differences in terms of gene expression or pathway analysis were noted when untreated controls and vaccinated non-survivors were examined independently, at least within our restricted dataset. Few differentially expressed genes (DEGs) were noted before 6 DPI possibly due to the delayed establishment of disease following a low dose challenge. Thus, the early stage was excluded from our analyses. Principal component analysis (PCA) revealed a clustering of fatal group samples at mid- and late-disease indicating our normalization method was satisfactory and that disease manifests similarly in rVSV-treated fatal rhesus macaques as it does for the untreated and vector controls (Fig. S3a).

### Survivor transcriptomes are characterized by early expression of interferon-related genes and Th1/Tfh-associated STAT4

Protective correlates mediated by the ∆G and N2 vectors were first determined by comparing whole blood transcriptomes of survivor and fatal samples using RNAseq. Only human homolog and protein-coding genes with a minimum reads per kilobase per million (RPKM) value of 5, a false discovery rate (FDR)-adjusted p-value of ≤0.05, and ≥ log_2_ fold change (FC) were included for further analysis.

**Figure 2:**
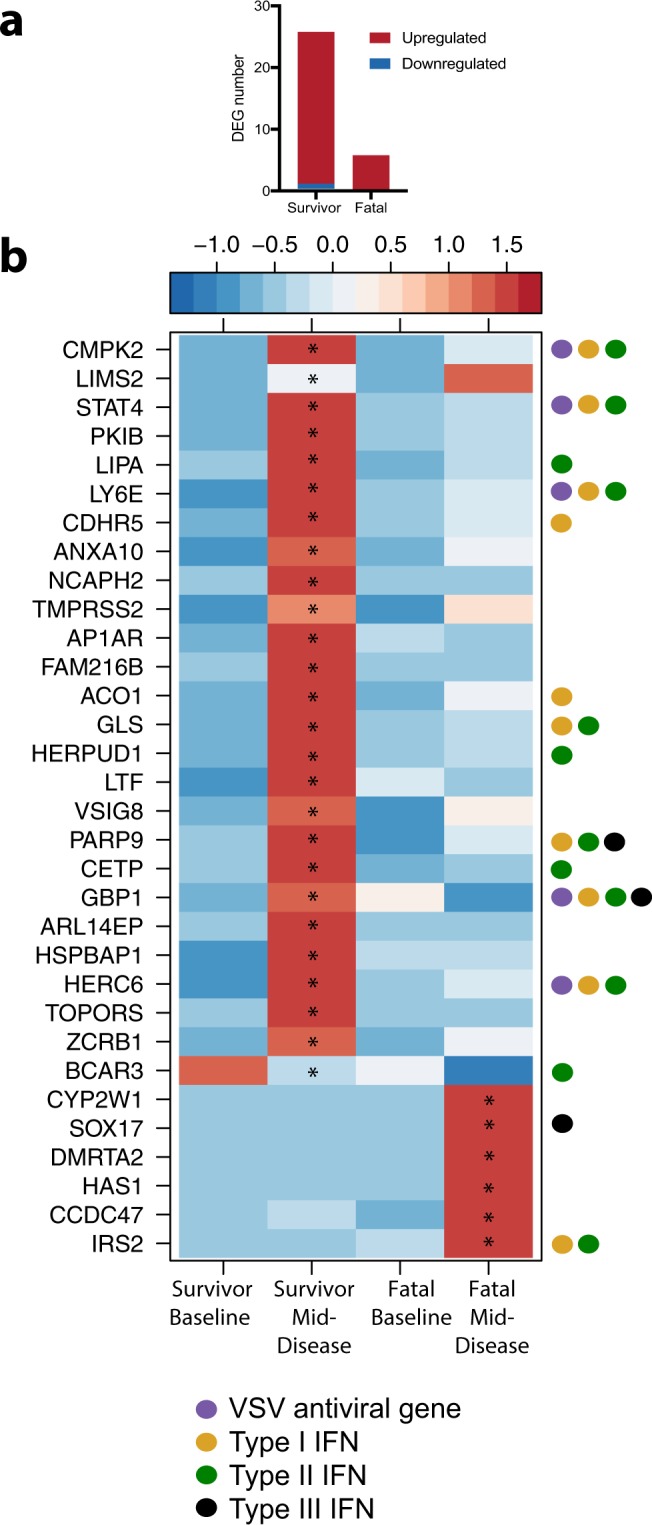
Upregulated and downregulated DEGs at mid-disease. DEGs were calculated using EdgeR against a pre-challenge baseline. (**a)** Bar graph of detected human homolog and protein coding DEGs. (**b)** Heatmap of all DEGs observed at mid-disease. A scaled heatmap based on reads per kilobase per million (RPKM) values within that set of genes (red represents increased expression while blue represents decreased expression); each column represents the median RPKM values for each time point. Genes were queried using the Interferome v2.01 database. Only human homologs and protein-coding genes were analyzed. *Statistically significant FDR-corrected p-value of ≤0.05.

At mid-disease, we identified 26 DEGs in survivors and 6 DEGs in fatal subjects relative to pre-infection with no shared genes (Fig. 2a). Nearly half of the DEGs detected in survivors at mid-disease were involved in or affiliated with interferon (IFN) signaling (Fig. 2b). Some upregulated IFN-stimulated genes (ISGs) in this dataset have demonstrated antiviral activity against VSV and other viruses *in vitro*, including *CMPK2*, which is a component of the nucleotide synthesis salvage pathway and contributes to terminal differentiation of monocytes; *LY6E*, which is affiliated with T-cell development; *HERC6*, which encodes a ubiquitin ligase functionally related to MHC-I class-mediated antigen processing and presentation; and *GBP1*, which promotes oxidative killing and delivery of antimicrobial peptides to phagolysosomes^29–35^.

Using the Interferome version 2.1 database, survivor ISG and IFN-affiliated genes were further examined to ascertain the dominant IFN subtype^36^. The majority mapped to type II IFN (IFN-gamma) signaling, although several genes were mutually shared with type I and III IFN subtypes. Type II IFN signaling was an interesting finding given the chief effector cytokine of Th1 cells is IFN-gamma, and we observed upregulation of *STAT4*, an early transcription factor that is associated with the differentiation of Th1 and T follicular helper (Tfh) cells^37,38^.

In the fatal dataset, fewer transcriptional changes were observed at mid-disease (Fig. 2a). Although *SOX17* and *IRS2* are constituents of the interferome and were upregulated in non-survivors, these genes are typically downregulated in the IFN response. The lack of ISG expression suggests a delayed or suppressed innate immune response in fatal cases.

### Poor prognosis is associated with gene signatures of apoptosis, immunosuppression, and Th2 responses at late-disease

At late-disease, robust gene expression changes were noted in fatal cases. Of the 4,576 DEGs that mapped to human homologs, 4,358 were upregulated and 218 were downregulated. In comparison, only 3 downregulated DEGs were detected in survivors. A heatmap of the most highly upregulated and downregulated DEGs is depicted in Fig. S4. Due to the abundance of DEGs for the fatal dataset, gene enrichment was performed using MetaCore to identify potential biomarkers and immune pathways associated with poor outcome.

Downregulated DEGS in fatal animals enriched to pathways and gene ontology (GO) annotations important for host defense, such as dendritic cell migration, toll-like receptor (TLR) signaling, CXCR4-mediated chemotaxis, and April and Baff signal transduction (Table 2). CXCR4 is a potent chemokine receptor that mediates lymphocyte chemotaxis, whereas April and Baff proteins augment immunoglobulin secretion and promote differentiation and proliferation of B-cells^39^. Another notable observation was decreased expression of DEGs mapping to the GO term “response to IL-12”, as this cytokine drives Th1 differentiation and we were unable to detect Th1-affiliated IFN-gamma or IL-2 reads in the fatal group at late-disease^40^.

**Table 2:** Enrichment analysis of DEGs at terminal disease in the fatal dataset.

Enrichment Pathway	Up/Down-Regulation	Metacore Enrichment Type	DEGs	FDR
Dendritic cell migration	Down	GO Processes	31	5.51E-03
TRIF-dependent toll-like receptor signaling pathway	Down	GO Processes	35	7.75E-03
Positive regulation of innate immune response	Down	GO Processes	430	1.24E-02
Interleukin-12-mediated signaling pathway	Down	GO Processes	88	1.93E-02
Response to interleukin-12	Down	GO Processes	90	2.06E-02
Apoptosis and survival_APRIL and BAFF signaling	Down	Pathway Maps	39	2.56E-02
Chemotaxis_CXCR4 signaling pathway	Down	Pathway Maps	34	4.88E-02
Apoptotic process	Up	GO Processes	1310	1.97E-15
Cell death	Up	GO Processes	1527	6.29E-15
Response to wounding	Up	GO Processes	924	2.30E-08
Response to transforming growth factor beta	Up	GO Processes	265	6.57E-08
Regulation of alpha-beta T cell differentiation	Up	GO Processes	105	3.88E-02
WNT signaling pathway	Up	GO Processes	511	2.33E-10
Protein folding and maturation_POMC processing	Up	Pathway Maps	30	1.46E-05
Development_WNT5A signaling	Up	Pathway Maps	47	3.16E-06
Immune response_Function of MEF2 in T lymphocytes	Up	Pathway Maps	51	9.54E-04
Immune response_IL-6 signaling pathway via JAK/STAT	Up	Pathway Maps	72	1.68E-03
Immune response_IL-4 signaling pathway	Up	Pathway Maps	94	4.08E-03
Immune response_IL-9 signaling pathway	Up	Pathway Maps	36	2.50E-03
Immune response_IL-5 signaling via JAK/STAT	Up	Pathway Maps	57	4.92E-03
Immune response_TLR2 and TLR4 signaling pathways	Up	Pathway Maps	69	7.11E-03
Immune response_IFN-alpha/beta signaling via MAPKs	Up	Pathway Maps	77	5.77E-03
Immune response_CRTH2 signaling in Th2 cells	Up	Pathway Maps	44	1.27E-02
Immune response_IFN-alpha/beta signaling via PI3K and NF-kB pathways	Up	Pathway Maps	94	1.38E-02
Immune response_CCL2 signaling	Up	Pathway Maps	54	2.55E-02
Normal and pathological TGF-beta-mediated regulation of cell proliferation	Up	Pathway Maps	33	4.13E-02
Immune response_Inhibitory PD-1 signaling in T cells	Up	Pathway Maps	53	4.17E-02

Selected enrichment terms listed were acquired using MetaCoreTM software (Thomson Reuters). Only protein-coding human homologs were included in these analyses. Abbreviations: FDR, false discovery rate-adjusted p-value; GO, gene ontology. All terms listed met the FDR-corrected p-value threshold of ≤ 0.05.

Upregulated DEGs in the fatal dataset enriched to pathway and GO terms associated with apoptosis and wound healing. Not until late-disease did we detect innate immune activation and induction of ISGs. An accumulation of transcripts mapping to IL-4, IL-5, IL-9, and CRTH2 signaling terms were also detected suggesting a T helper 2 (Th2)-skewed response. CRTH2 is considered a reliable marker for circulating Th2 cells and stimulates chemotaxis of these cells^41^. Two key transcription factors that drive Th2 polarization in naïve T-cells include *STAT6* (log_2_FC: 4.82; FDR: 4.90E-04) and *GATA3* (log_2_FC: 3.02; FDR: 1.95E-02), both of which were significantly upregulated in the fatal dataset^42,43^. In line with our enrichment analysis pointing to IL-9 signaling in the fatal dataset, we also observed increased expression of transcriptional regulators associated with Th9 development including *IRF4* (4.17 log_2_FC; FDR: 1.81E-03), *STAT6* (4.82 log_2_FC; FDR: 4.90E-04), *GATA3* (3.02 log_2_FC; FDR: 1.95E-02), *PU.1* (5.03 log_2_FC; FDR: 1.88E-03), *NFAT1* (4.58 log_2_FC; FDR: 4.08E-04), and *AP-1* (4.71 log_2_FC; FDR: 1.55E-03)^44^.

Many upregulated transcripts in non-surviving animals at late-disease mapped to processes associated with immunoregulatory signaling including transforming growth factor-beta (TGF-beta), programmed cell death protein 1 (PD-1), and Wnt signaling pathways. TGF-beta is a multifunctional cytokine that stimulates apoptosis, inhibits T- and B-cell proliferation, and mediates the polarization of naïve and effector T-cells to regulatory T-cells (Tregs)^45^. Provided Tregs negatively regulate immune and T-effector responses by upregulating inhibitory receptors, such as PD-1, lymphocyte activation gene 3 (LAG-3), and cytotoxic T-lymphocyte-associated protein 4 **(**CTLA-4), we also examined the expression of these immune checkpoint molecules^46^. In fatal animals, the number of DEGs enriching to “Immune response_Inhibitory PD-1 signaling in T cells” pathway was increased, along with the genes *PD-1* (3.09 log_2_FC; FDR: 1.69E-02) and *LAG-3* (4.17 log_2_FC; FDR: 1.84E-03), but not *CTLA-4*. Interestingly, two of the most highly expressed DEGs in the fatal dataset encoded *Wnt5A* (5.99 log_2_FC; FDR: 3.07E-04) and *Wnt6* (7.78 log_2_FC; FDR: 7.77E-05). Wnt5a was shown to promote the differentiation of dendritic cells to a tolerogenic state by increasing indoleamine 2,3-dioxygenase-1 (IDO) activity and decreasing IL-12, HLA-DR, and costimulatory molecule (CD80, CD83, and CD86) expression^47,48^. Wnt6 is thought to act on neighboring macrophages to induce proliferation and polarization toward an anti-inflammatory M2 phenotype^49^.

### Th2 and CD8+ T-cell-associated transcripts correlate with lethality

**Figure 3:**
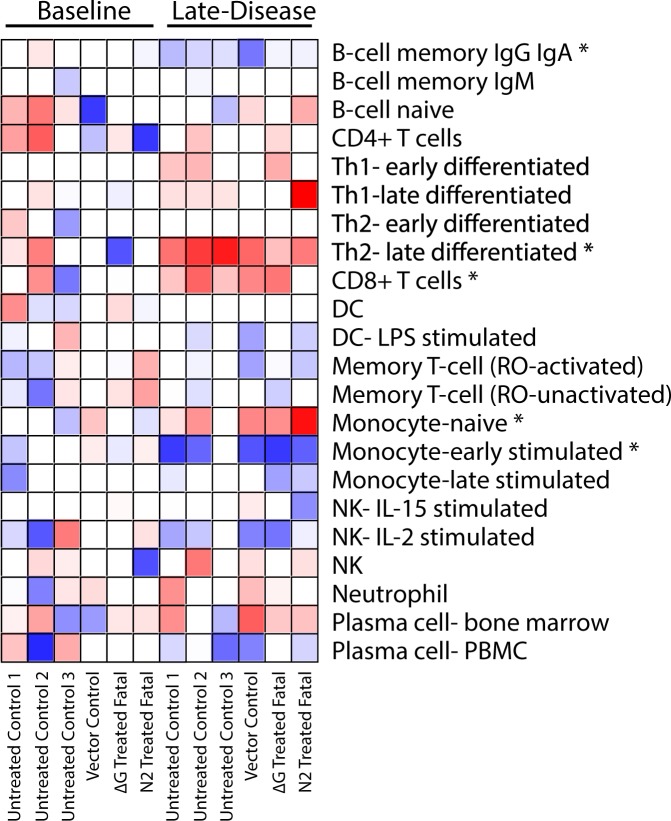
ImmQuant heatmap analysis of the relative contribution of immune cell subsets to differential gene expression. Results were calculated using ImmQuant, a database based on genome-wide microarray expression profiling of human immune cells, and the IRIS algorithm that incorporates human-based FACS marker genes. The algorithm infers an increase (red) or decrease (blue) in cell-type quantities relative to a pre-challenge baseline. *Statistically significant (FDR-adjusted p-value ≤ 0.05) putative change in the cell subset frequency.

Next, we performed digital cell quantification (DCQ) on the fatal dataset to predict the relative contribution of specific immune cell subsets to differential gene expression^50,51^. RNAseq transcript abundance at late-disease was compared to a pre-challenge baseline. This analysis predicted a statistically significant increase in the frequency of late-differentiated Th2- and CD8+ T-cells in non-survivors (Fig. 3). These observations reflect the extensive Th2-associated signaling observed following functional enrichment with MetaCore. In contrast, our DCQ analysis predicted an increase in naïve monocytes and a decrease in the proportion of IgG/IgA memory B-cells and early-stimulated monocytes. As monocytes are early and sustained target cells for MARV infection, the immature subset may have served to disseminate infection^52^. When the analysis was repeated using differentially expressed transcripts for the untreated controls only, we also found a significant reduction in frequencies of Th1 and stimulated dendritic cells (Fig. S5).

### Survivor responses are Th1-skewed, whereas fatal responses are associated with Treg and/or Th2 cell recruitment

**Figure 4:**
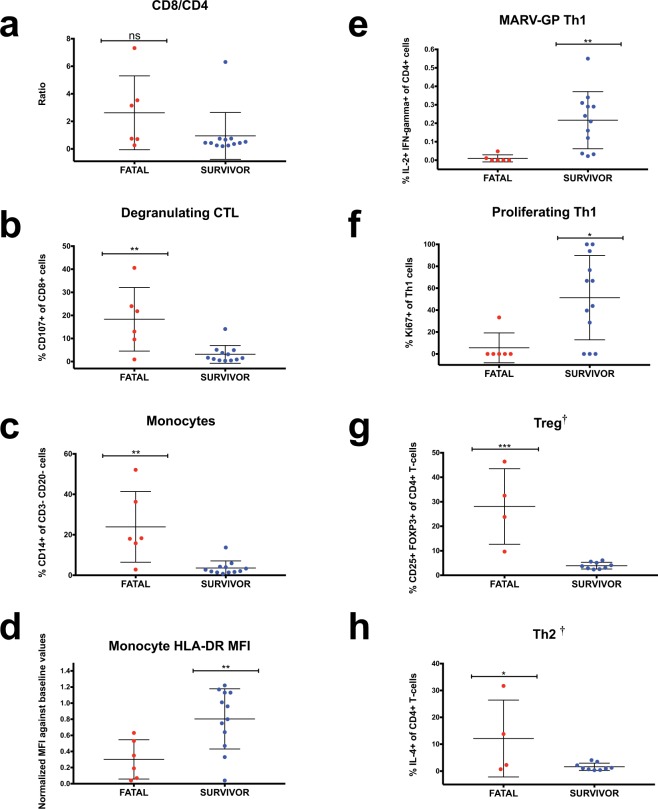
Analysis of macaque PBMCs using flow cytometry. (**a)** CD8+/CD4+ ratio of T-lymphocytes (CD3+ cells) for individual fatal (red; N = 6) and survivor (blue; N = 12) macaque subjects. (**b)** Percentage of degranulating T-cells based on CD107a expression within the CD8b+ subset. (**c)** Percentage of monocytes (CD3-CD20-CD14+ cells). **(d)** HLA-DR expression of monocytes based on relative mean fluorescence intensity (MFI) value. (**e)** Frequency of polyfunctional (IFN-gamma+ IL-2+) Th1 cells. (**f)** Frequency of proliferating Th1 (Ki67+) cells. (**g)** Frequency of regulatory T-cells (Tregs; CD3+CD4+CD25+FOXP3+). (**h)** Frequency of Th2 cells (CD3+CD4+IL-4+). Two-tailed t-test *p ≤ 0.05; **p ≤ 0.01; ***p ≤ 0.001; ****p ≤ 0.0001; ns, not significant. †Denotes historical samples were not included in the analysis (remaining N = 4 for fatal group and N = 9 for the survivor cohort).

To confirm whether our sequencing results and cell subset projections were reflected in the blood compartment at late-disease, we performed flow cytometry on peripheral blood mononuclear cells (PBMCs). PBMCs were taken from identical sampling time points as those used for transcriptomic analysis. In contrast to our DCQ results, we did not detect a higher CD8/CD4 proportion in fatal cases (Fig. 4a). However, a more substantial percentage of degranulating (CD107+) CD8+ CTLs were found in non-survivors (Fig. 4b). Additionally, we determined the percentage of monocytes (CD3-CD20-CD14+) and discriminated their maturation state by examining expression of HLA-DR (an MHC II molecule). Our data revealed an increase in total monocytes in fatal subjects (Fig. 4c), but augmented HLA-DR expression in survivors (Fig. 4d), corroborating our DCQ analysis. This suggests lethality might be associated with recruitment of immature monocytes. Finally, as we postulated *STAT4* expression might induce Th1 differentiation in survivors, we performed intracellular cytokine staining at late-disease. PBMCs were stimulated overnight with an overlapping MARV-GP peptide pool. As expected, the frequency of polyfunctional and proliferating antigen-specific IL-2- and IFN-gamma-secreting Th1 cells were higher in survivors compared to fatal animals (Fig. 4e,f).

Given specific gene signatures pointed to differentiation of Tregs, we hypothesized these cells were recruited in fatal cases. While Tregs have been implicated in MARV persistence in the testes of NHPs^53^, this is the first study to report recruitment of this T-cell subset in the blood compartment and in the context of acute filovirus disease. For this analysis, we were unable to include subjects from the first historical experiment due to insufficient sample availability. Tregs in the remaining samples (N = 4 fatal subjects; N = 9 treated survivors) were identified based on CD25+ FOXP3+ expression within the CD3+ CD4+ T-cell population. Tregs normally account for 5–10% of circulating T-cells and are responsible for immune tolerance and prevention of autoimmunity^54^. In non-surviving subjects at late-disease, this subset accounted for up to ~40% of all CD4+ T-cells (Fig. 4g). Within the Treg subset, we confirmed secretion of the prototypical cytokine, IL-10 (Fig. S6b). An antibody against IL-4 was additionally included in the panel to discriminate Th2 populations. In accordance with our gene enrichment and DCQ analyses, the frequency of Th2 cells was increased in fatal cases, although some heterogeneity existed within the group (Fig. 4h).

### Survivors express higher levels of NK cells and a subset (CD16+) associated with higher cytotoxicity at late disease

**Figure 5:**
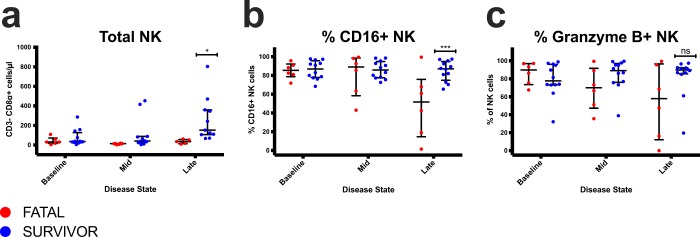
Analysis of NK cell populations in macaque PBMC at each disease stage. (**a)** Absolute total NK cell counts. (**b)** Percentage of CD16+ NK cells within the CD8α+ (NK) subset. (**c)** Percentage of granzyme B+ cells within the CD8α+ (NK) subset. Multiple two-tailed t-tests *p ≤ 0.05; ***p ≤ 0.001; ns, not significant. Individual fatal (red; N = 6) and survivor (blue; N = 12) macaque subjects.

Since others have demonstrated the importance of NK cells in vaccine-mediated protection with Ebola virus-like particles^55^ or an EBOV GP-based rVSV^56^, we also explored whether NK cells or specific subsets were critical for survival in immunized NHPs after infection. Similar to these reports, we found that survivors had a higher proportion of total NK (Fig. 5a) and CD16+ NK cells (Fig. 5b) at late disease, but not granzyme B+ (Fig. 5c) NK cells.

### Non-survivors have a dysregulated pro- and anti-inflammatory balance

**Figure 6:**
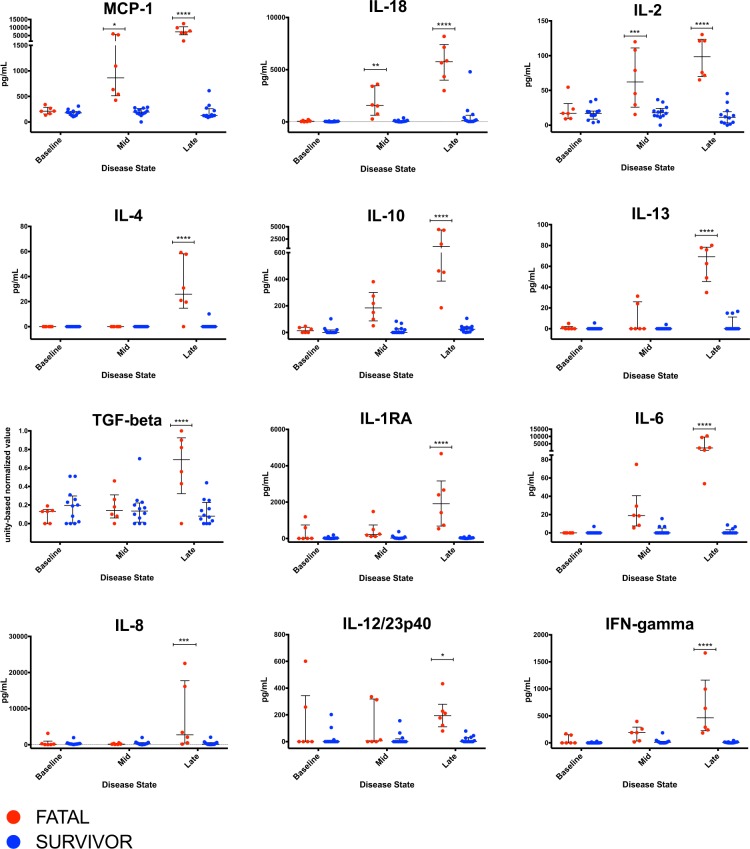
Plasma levels of cytokines/analytes in macaque subjects. Red dots represent individual macaques in the fatal dataset; blue dots represent individual macaques in the treated survivor dataset. *p-value ≤ 0.05; **p-value ≤0.01; ***p-value ≤ 0.001; ****p-value ≤ 0.0001. Two-way ANOVA followed by a Bonferroni multiple comparisons test (alpha = 0.05).

To evaluate the systemic immune response in each group, plasma cytokine concentrations were measured at baseline, mid- and late-disease using cytokine bead arrays (Fig. 6). Early expression of pro-inflammatory chemoattractants (MCP-1, IL-18, IL-2) coincided with onset of clinical disease in fatal cases, followed by late systemic increases in Th2 (IL-4, IL-10, IL-13), anti-inflammatory (TGF-beta, IL-10, IL-1RA), and other Th1-associated/pro-inflammatory (IL-6, IL-8, IL-12, IFN-gamma) mediators. In treated survivors, concentrations of these cytokines and chemokines remained similar to pre-challenge values.

## Discussion

In this study, we employed a combination of flow cytometry, multiplex bead arrays, and RNA sequencing to uncover immune correlates of post-exposure protection conferred by rVSV vectors expressing MARV GP following a low dose lethal challenge. Our analysis revealed innate immune activation, antibody production, and T-cell responses likely acted together to elicit protection against MVD in treated survivors.

The timing and magnitude of the type I IFN response may be a crucial factor for protection against MVD given rapid upregulation of ISGs was noted in survivors. MARV proteins antagonize ISG induction early in infection, resulting in unrestricted viral growth, aberrant innate immunity, and deferment of adaptive immunity. MARV viral protein 40 (VP40) is known to interfere with the JAK/STAT pathway by blocking the phosphorylation of JAKs to prevent type I and II signaling, and viral protein 35 (VP35) antagonizes RIG-I/MDA-5 signaling^57,58^. Activation of innate immune signaling by rVSV-MARV-GP likely curbs MARV viral replication to allow sufficient time for generation of an adaptive response. However, MARV may circumvent these innate responses in certain hosts. This hypothesis is supported by the fact that viral load was lower in treated survivors in this study and only these animals formed antigen-specific IgG and developed polyfunctional Th1 cells.

Besides virus-specific antibodies and Th1 cells, we also detected a higher proportion of total NK and CD16+ NK cells in treated survivors. This finding may be of importance since NK cells were also critical for vaccine protection against Ebola virus disease for virus-like particle and rVSV platforms. Two phenotypically and functionally distinct peripheral blood NK cell subsets have been described in humans based on their expression of CD56 and CD16^59^. The predominant CD56^dim^ population expresses Fc-gamma receptor III (CD16) and is known for its increased cytolytic activity, limited cytokine production, and enhanced antibody-dependent cell-mediated cytotoxicity (ADCC) activity. In contrast, the less frequent CD16^−^CD56^bright^ NK cell subset is considered to possess regulatory functions. Fritz *et al*. previously demonstrated that CD16+ NK cells declined in peripheral blood in fatal MARV-Ci67 infection^60^ and Williams *et al*. showed NK cell-mediated cytotoxicity was significantly higher following VSVΔG/EBOV GP immunization^56^. Thus, CD16+ NK cells may contribute to rVSV-mediated postexposure protection by killing virally infected cells or initiating ADCC.

A non-specific vector response alone does not appear to drive postexposure protection as an irrelevant rVSV vector does not enhance survival in this study or others^15,18^. While the non-specific vector used in this study is highly attenuated (by relocating the N-gene to the fourth genomic position) and accordingly cannot be directly compared with the ∆G and N2 vectors, the references cited prove non-attenuated Ebola GP- or Lassa GP-expressing rVSVs (∆G vectors) do not provide postexposure protection against MVD either. However, non-specific vectors might delay the time-to-death presumably due to activation of innate immunity. Our findings contradict a previous report demonstrating that rhesus macaques injected with 2 doses of rVSV∆G/MARV-Angola-GP at 1 and 24 hours postexposure were partially protected against lethal EBOV challenge, suggesting nonspecific responses drive rVSV-mediated immunity^20^. The number of treatment doses (2) in this study, or the fact that EBOV is not uniformly lethal in rhesus macaques, might explain this discrepancy. For future studies, a larger vector control cohort will provide more meaningful results.

Our data revealed robust induction of anti-MARV IgM/IgG in treated survivors and low PRNT_50_ values. These results indicate non-neutralizing mechanisms of antibody-mediated viral clearance, e.g. opsonization, that likely contribute to protection against MARV-Angola. In contrast, anti-MARV IgM and IgG titers and neutralizing antibodies were either low or undetectable in fatal cases. MVD-induced interference of signaling molecules within the April and Baff pathway may have hindered antibody and B-cell development given transcripts enriching to this pathway were diminished in fatal cases. April and Baff proteins are potent coactivators that augment Ig production and help upregulate B-cell effector molecules^39^. DCQ analysis predicted a decrease in the proportion of IgG/IgA memory B-cells, which may have additionally impaired humoral responses.

Polarization towards the Th1 effector subset in treated survivors may reflect tightly coordinated or localized secretion of IL-12 by antigen-presenting cells early in infection, as we did not detect a systemic increase of this cytokine in these subjects. Accordingly, minimal transcriptional changes in the survivor dataset were observed at late-disease. In response to IL-12, Th1 cells secrete IFN-gamma and IL-2 to stimulate antigen presentation, cellular immunity, and production of IgG opsonizing antibodies^37,40^. *STAT4* expression can also foster differentiation of Th1 and/or Tfh cells. Tfh cells secrete IL-21 following antigenic stimulation and migrate into B-cell follicles to provide cognate help to germinal center B-cells^37,38^. Thus, *STAT4*-induced Th1 and Tfh cells may contribute to rVSV postexposure protection by promoting cellular and humoral responses, respectively.

Stonier *et al*. demonstrated that natural survival during the 2012 MARV outbreak in Uganda was dependent on Th1 immunity in humans^61^. Th2-skewed immunity seen in fatal subjects in this study may enhance MVD by promoting secretion of the Th2-associated cytokines IL-4 and IL-10, thereby suppressing Th1 production of IFN-gamma and IL-2^62^. Th2 cells are typically triggered by IL-4 secretion and are important for humoral immunity (secretion of IgE and certain IgG subtypes) as well as defense against extracellular parasitic infections^40^. The primary functions of Th2 cells are to stimulate B-cells to produce IgE antibodies and activate eosinophils, basophils, and mast cells. A common theme for many pathogenic infection scenarios is the promotion of a host Th1/Th2 imbalance that favors replication of the organism. While a Th2 shift serves to suppress an overactive pro-inflammatory response and ameliorate tissue destruction, it can have a profound effect on the susceptibility of a host to disease or infection.

Connor, Lin, *et al*. were the first to describe a commitment to the Th2 lineage in the context of MVD^27,63^. MARV-Angola infection in cynomolgus macaques led to early Th2-associated IL-4 and IL-5 signaling and increasing plasma concentrations of IL-10. No appreciable changes in Th1 cytokines, such as IL-12 and IFN-gamma, were found until late in disease, which is consistent with our reports. From these results, the authors concluded delay of Th1 immunity ultimately contributed to tissue damage and immunopathology, as the response was too late to combat infection. Presumably, this may explain why degranulating CD8+ T-cells found in fatal cases in this study also did not ameliorate disease. Another hypothesis is that CTLs are unable to recognize virally infected cells as antigen presentation is blunted in these individuals. In this study, we noted a statistically significant decrease and increase in the frequency of DCQ-predicted stimulated and naive monocytes, respectively. Naïve monocytes express lower levels of co-stimulatory molecules, which may not adequately support T-cell activation^64^. Alternatively, these recruited “immature” monocytes could represent myeloid-derived suppressor cells (MDCS). These cell populations closely resemble monocytes and granulocytes, express low levels of HLA-DR and the monocyte marker, CD14, and expand during chronic and acute inflammatory conditions^65^. Induction of these cells is mediated by growth factors, IL-6, and IL-10, which were abundantly expressed in animals that succumbed. MDSCs suppress immune effector activity via various mechanisms, including the production of reactive oxygen, secretion of immunosuppressive cytokines like IL-10 and TGF-beta, and by activation of Tregs.

Although an anti-inflammatory immune milieu prevents autoimmunity and mitigates tissue damage, consequences of this phenotype include loss of T-cell effector function, sustained upregulation of inhibitory receptors, and secretion of immunosuppressive cytokines, all of which promote viral growth^66,67^. Given we observed Wnt/inhibitory PD-1 signaling and recruitment of Tregs in fatal cases, MARV may have evolved mechanisms to promote a tolerogenic/immunoregulatory transcription program that promotes disease. Increasing evidence suggests a regulatory function of Wnt proteins in inflammation, cancers, and infectious diseases. Other pathogens such as *Mycobacterium tuberculosis*, influenza, Hepatitis B virus, and certain bunyaviruses are known to manipulate the host Wnt network to enhance immune evasion and replication^68–72^. Specifically, we observed high expression of genes encoding the Wnt proteins, Wnt6 and Wnt5a, in fatal subjects. Wnt6 is known to polarize macrophages to an M2 phenotype. M2 macrophages produce high levels of IL-10 and TGF-beta, and low levels of IL-12^49^. Provided we observed increased upregulation of DEGs enriching to IL-4 signaling and decreased transcripts mapping to IL-12 signaling in fatal cases, MARV infection may have instigated interaction with Wnt pathway elements to foster disease. Furthermore, Wnt5a-induced expression of the immune checkpoint regulator, IDO, may generate myeloid-derived suppressor cells and activate Treg expansion^47^. Enhanced IDO activity can also suppress effector T-cell activation and direct successive expansion of Tregs^48^.

Tregs negatively regulate immune and T-effector responses to protect the host against inflammation-mediated immunopathology, but also impede pathogen clearance. To prevent damage, Tregs secrete immunosuppressive cytokines such as IL-10, TGF-beta, and IL-35, and upregulate inhibitory receptors, such as PD-1^46^. PD-1 dampens an overactive immune response via two methods: fostering apoptosis of antigen-specific T-cells and reducing apoptosis of Tregs. In addition, secretion of inhibitory IL-10 by Tregs is known to prompt downregulation of HLA-DR expression in monocytes interfering with humoral immunity. Polarization of naïve T-cells towards this subset may result in a suboptimal immune response against MVD. Ruibal *et al*. showed that CD4+ and CD8+ T-cells from fatal cases during the 2013–16 EBOV epidemic in West Africa had lower expression of PD-1 and immunosuppressive IL-10, illustrating the importance of these immunoregulatory molecules in filovirus pathogenesis^73^.

One limitation of this study is that we did not determine whether circulating Th1, Th2, or Treg cells were actually recruited to secondary lymphoid organs, or if the Th2 response was antigen-specific. Moreover, Th2 cells were not identified among all subjects within the fatal group. This may be explained by commitment to other Th lineages that we did not analyze by flow cytometry. Recently it was shown that the Th2 cytokines IL-4 and IL-9 are seldom produced by the same T-cell indicating another discrete IL-9-producing subset exists: Th9 cells^44^. TGF-beta induction causes Th2 cells to convert to Th9 cells, suggesting there is some plasticity among these subsets that is primarily influenced by the cytokine environment. Besides IL-9, Th9 cells secrete IL-17, IL-21 and IL-22. Physiological and pathophysiological roles of the Th9 sub-population are still not entirely clear, but these cells have been implicated in both protective (tumor suppression and parasitic infections) and pathogenic (allergy, colitis, autoimmunity and fibrosis) functions. TGF-beta and IL-4 are indispensible for Th9 polarization and IL-2 strongly enhances development. Along with our enrichment analysis that pointed to TGF-beta, IL-4, and IL-9 signaling, we also observed upregulation of numerous transcriptional regulators that support Th9 development.

Another caveat is that only whole blood was examined for our RNAseq analysis. T- and B-cell populations represent a minority of cells in peripheral blood, which may have precluded us from identifying additional immune signatures associated with protection. Purification of CD20+ (B-cell), CD4+, and CD8+ populations or single cell RNA-seq might yield more relevant results in the future, particularly since we were unable to detect B-cell signatures in the survivor group despite the presence of antibodies.

Collectively, these data indicate well balanced and concerted pro-inflammatory/anti-inflammatory interactions are likely pivotal for immunity against MVD. Impaired immunoregulation and upregulation of immune checkpoint molecules are associated with poor prognosis regardless of treatment. Early *STAT4* and IFN signaling following rVSV treatment may play a role in establishing Th1/Tfh immunity and antibody production to elicit protection in survivors. Combination therapy with Th1/Tfh-inducing adjuvants might enhance rVSV postexposure protection against this deadly virus, particularly in non-responders.

## Methods

### Generation of rVSV vectors

rVSV vectors were recovered from infectious clones. An expression cassette encoding the full-length Angola glycoprotein (MARV-Angola-GP, accession number: DQ447653) was cloned into plasmids containing the entire Vesicular stomatitis virus (VSV) genome. To generate rVSV∆G/MARV-Angola-GP (the “∆G” vector), a PCR-amplified Angola GP gene was cloned into the Mlu I/Nhe I gene site in place of the native VSV glycoprotein (G) gene. The resulting plasmid was then transfected into a BHK-21 cell line (ATCC cat: CCL-10). These cells were previously transfected with VSV G and infected with a Vaccinia virus that constitutively expresses T7 polymerase. The plasmid contained T7 polymerase promoter and terminator sequences at the 3′ and 5′ ends of the rVSV genome to drive gene expression. Nucleoprotein (N), phosphoprotein (P), and polymerase (L) helper plasmids were co-transfected into the infected cells to promote recovery. Recovered virus supernatants were subsequently filtered to remove contaminating Vaccinia virus and passaged on Vero cells. The amplified virus was then plaque-purified and passaged a second time.

The remaining vectors were engineered at Profectus BioSciences, Inc. To attenuate rVSVN4CT1-HIVgag (the “vector control”), the VSV N gene was translocated from the first to the fourth (N4) genomic position and the VSV G cytoplasmic tail (CT1) was truncated. To produce rVSVN2CT1-MARV-Angola-GP (the “N2” vector), the VSV N was shuffled from the first to the second (N2) genomic position. The GP gene was expressed at the first position to effectively drive GP antigen expression.

### Challenge virus

The MARV Angola seed stock originates from the 2004–2005 Uige, Angola outbreak (DQ: 447653.1). The virus was isolated from the serum of a fatal patient on March 13, 2005 (8-month old female; isolate 200501379). The study challenge material was created by passaging the original isolate twice in Vero E6 cells (titer 1.5 × 107 PFU/mL). Endotoxin and mycoplasma contamination were not detected in stocks.

### Animal study design

Eighteen adult (13 females and 5 males) rhesus macaques (*Macaca mulatta*) from PrimGen Labs were randomly assigned to each cohort. Animal weights ranged from 3.6 to 5.6 kg. All macaques were i.m. challenged in the left quadriceps with a low uniformly lethal target dose of 50 PFU (actual dose was 45–80 PFU) of MARV Angola. Approximately 20–30 minutes after exposure, subjects within the ∆G (N = 9) and N2 (N = 5) treatment groups were administered a single 10 million PFU i.m. injection of each rVSV vector. The inoculation was equally distributed between the left and right quadriceps. Four of the nine subjects that received the ∆G vector were published in a previous study, along with the non-specific vector control (N = 1) and untreated controls (N = 3)^16^. However, Untreated Controls 2 and 3 were challenged simultaneously with the remaining ∆G (N = 5) animals, whereas the vector control was challenged at the same time as the N2 (N = 5) treated animals. An internal scoring protocol was implemented to track disease progression in challenged animals, and included criteria such as posture/activity level, appetite, behavior, respiration, and the presence of hemorrhagic manifestations. Animals were checked at least twice daily. Subjects that reached a clinical score ≥ 9 were euthanized with a pentobarbital solution. Blood samples were taken temporally at 0, 3, 6, 10, 14, 21, and 28 post-challenge, or terminally. Major organ tissues were isolated at the study endpoint to determine viral titers. These studies were not blinded.

### Ethics statement

Animal studies were performed in BSL-4 biocontainment at the University of Texas Medical Branch (UTMB) and approved by the UTMB Institutional Biosafety Committee (IACUC). Animal research was conducted in compliance with UTMB IACUC, Animal Welfare Act, and other federal statutes and regulations relating to animals. The UTMB animal research facility is fully accredited by the Association for Assessment and Accreditation of Laboratory Animal Care.

### Blood processing and PBMC isolation

Blood was collected by femoral venipuncture into EDTA, heparin, and clot activating vacutainer tubes (BD Biosciences, San Jose, CA). An aliquot of EDTA-treated whole blood was diluted 1:6 with AVL inactivation buffer (Qiagen, Hilden, Germany) for RNAseq and RT-qPCR analyses. The EDTA plasma and serum tubes were centrifuged at ~800 × g for 10 minutes; afterward, the upper layer was collected. For isolation of PBMCs, heparin-treated blood and the spun EDTA pellet were diluted with PBS and carefully layered onto a Histopaque cushion within Accuspin tubes (Sigma, St. Louis, MO). The tubes were centrifuged at ~800 × g room temperature (RT) for 15 minutes and the resulting buffy coat was collected. Cells were washed once in R10 (RPMI media (Gibco, Gaithersburg, MD) supplemented with 10% fetal bovine serum (FBS), 100 U/mL penicillin, 100 g/mL streptomycin solution, and 1% L-glutamine) and treated briefly with ACK lysing buffer (Gibco, Gaithersburg, MD) to remove any contaminating erythrocytes. PBMCs were then centrifuged at ~250 × g for 10 minutes to eliminate residual platelets, washed twice with R10 media, and enumerated with a TC20 Automated Cell Counter (Bio-Rad, Hercules, CA). Cells were cryopreserved in 10% dimethyl sulfoxide (DMSO) in FBS. Before performing flow cytometry, cryopreserved PBMCs were thawed rapidly in a 37 °C water bath and washed in BD Staining Buffer (BD Biosciences, San Jose, CA).

### Hematology and serum biochemistry

EDTA-treated blood was analyzed using a laser-based hematologic analyzer (Beckman Coulter) to determine total white blood cell counts, white blood cell differentials, red blood cell counts, platelet counts, hematocrit values, total hemoglobin concentrations, mean cell volumes, mean corpuscular volumes, and mean corpuscular hemoglobin concentrations. A Piccolo point-of-care analyzer and Biochemistry Panel Plus analyzer discs (Abaxis) were used to test for serum concentrations of albumin, amylase, alanine aminotransferase (ALT), aspartate aminotransferase (AST), alkaline phosphatase (ALP), gamma-glutamyltransferase (GGT), glucose, cholesterol, total protein, blood urea nitrogen (BUN), creatinine (CRE), uric acid, and C-reactive protein (CRP).

### Virus plaque assay

MARV viremia was titrated by plaque assay on Vero E6 cells (ATCC Cat: CRL-1586). Briefly, increasing ten-fold dilutions of plasma samples were adsorbed to monolayers in duplicate, overlaid with 0.8% agarose/2x EMEM, and incubated for six days at 37 °C in 5% CO_2_. Neutral red stain was added and plaques were counted after a 48-hour incubation. The limit of detection for this assay is 25 PFU/mL.

### RNA extraction

RNA was extracted using a Qiagen Viral RNA Mini kit (Qiagen Mississauga, ON, Canada). AVL-treated whole blood was first lysed using Qiagen Qiashredder tubes to liberate intracellular RNA. RNA was extracted according to the manufacturer recommendations and treated with DNase.

### RT-qPCR virus copy determination

One-Step Probe RT-qPCR kits (Qiagen) and CFX96 system/software (BioRad) were used to determine viral copies in samples. To detect MARV RNA, we targeted the MARV NP gene with primer pairs and a 6-carboxyfluorescein (6FAM)–5′-CCCATAAGGTCACCCTCTT-3′–6 carboxytetramethylrhodamine (TAMRA) probe. Thermocycler run settings were 50 °C for 10 min; 95 °C for 10 s; and 40 cycles of 95 °C for 10 s plus 59 °C for 30 s. Integrated DNA Technologies synthesized all primers and Life Technologies customized probes. Representative MARV genomes were calculated using a genome equivalent standard. The limit of detection for this assay is 1000 copies/mL.

### Bead-based multiplex immunoassays

Concentrations of cytokines and other analytes were assayed using bead-based multiplex technology. Irradiated plasmas were incubated with magnetic beads from Milliplex NHP Cytokine Premixed 23-plex Panel (EMD Millipore, Billerica, MA) or ProcartaPlex NHP TGF-beta 1 simplex (eBioscience, Vienna, Austria) kits according to the manufacturer protocols. The concentrations in each plasma sample were measured using a Bioplex-200 array system (BioRad, Hercules, CA).

Plasma levels of coagulation-associated parameters were quantified using a flow-based Legendplex Human Thrombosis 7-plex bead immunoassay (Biolegend, San Diego, CA). Samples were processed in duplicate following the kit instructions to measure concentrations of the following analytes: p-selectin, d-dimers, p-selectin glycoprotein ligand-1 (PSGL-1), tissue plasminogen activator (tPA), soluble CD40-ligand (sCD40L), plasminogen activator inhibitor-1 (PAI-1), and factor IX. Approximately 2,100 events (beads) per sample were collected on a FACS Canto II cytometer (BD Biosciences, San Jose, CA) using BD FACS Diva software and the raw data was analyzed with BioLegend’s cloud-based LEGENDplex™ Data Analysis Software.

### RNA-seq library preparation

RNA concentration and quality were determined with Agilent 2100 Bioanalyzer using an Agilent RNA 6000 Nano Kit. RNA samples were purified with Agencourt RNAClean XP beads prior to library preparation. Illumina TruSeq Stranded Total RNA LT kit was used to deplete ribosomal RNA (rRNA) and construct cDNA libraries according to the instructions provided by the manufacturer. RNA was fragmented, converted to double-stranded cDNA, and adapters ligated to each strand. The resulting ~300 base-pair cDNA fragments were then amplified by PCR and purified using AMPureXP Beads. Each library was prepared with a unique indexed adapter for multiplexing. Libraries were validated for size, concentration, and integrity with Agilent 2100 Bioanalyzer using Agilent High Sensitivity DNA Kit. Multiplexed libraries were subjected to single-end 75 base pair sequencing using the Illumina NextSeq500 V2 platform.

### RNA-seq analysis and functional enrichment

The RNA-seq workflow module of Bioconductor’s systemPipeR open source software was used to perform the bioinformatic analysis. Sequences were demultiplexed using bcl2fastq conversion software. Quality control of sequences was assessed using the FastQC function. Trimmomatic was used to trim three base pairs from the 5′ end and two bases from the 3′ end. Only RNAseq reads with a phred score ≥ 30 and a 50 base pair minimum length were included. The *Macaca mulatta* genome sequence (Macaca_mulatta.MMUL_1.dna.toplevel.fa) and corresponding annotation file from Ensembl (Macaca_mulatta.MMUL_1.78.gtf) were implemented for alignment purposes. Bowtie2/Tophat2 suite was used to align cleaned and trimmed RNA-Seq reads to the viral and macaque reference genomes. The summarizeOverlaps function generated raw read counts that mapped to overlapping exon regions of genes and discarded reads that mapped to ambiguous exon regions. The edgeR function was executed to normalize DEGs against a pre-challenge baseline (0 DPI). DEGs were determined with ≥ log_2_ fold change (FC) and a false discovery rate (FDR) corrected p-value threshold of ≤ 0.05. Only genes encoding proteins with human homologs and an average of 5 RPKM were evaluated. Figures were generated with R tools and Adobe Illustrator. Heatmaps were created with VennDiagram and gplot package. We used MetaCore^TM^ (Thomson Reuters, New York, NY) to identify functionally related gene groups mapping to specific biological pathways. Interferon-stimulated genes (ISGs) were discovered using the Interferome v2.0 database. Cell-type quantity matrix and comparative viewer images were created using ImmQuant software and the IRIS algorithm^50,51^.

### Immunophenotyping and intracellular cytokine staining (ICS)

For the monocyte and NK cell flow panels, PBMCs were surface stained in the dark at 4 °C with fluorochrome-conjugated antibodies against CD3 (BD clone: SP34; RRID:AB_396484; FITC (both panels)), CD20 (BD clone: 2H7; RRID:AB_396501; FITC (both panels)), CD14 (Biolegend clone: M5E2; APC (monocyte panel, RRID:AB_314190) or FITC (NK panel, RRID:AB_314186)), HLA-DR (Biolegend clone: L243; RRID:AB_493586; APC/Cy7 (monocyte panel)), CD8a (Biolegend clone: RPA-T8; RRID:AB_314134; APC/Cy7 (NK cell panel)), CD16 (Biolegend clone: 3G8; RRID:AB_893262; PerCP/Cy5.5 (NK cell panel)), and granzyme B (Biolegend clone: GB11; RRID:AB_2566333; Alexa Fluor 647 (NK cell panel)). Cells were briefly centrifuged, washed with BD staining buffer (BD cat#: 554657), and fixed with a 4% paraformaldehyde solution. PBMCs were washed again before final resuspension in BD staining buffer.

To examine the polyfunctionality and frequency of antigen-specific Th1 and CTL populations, we performed ICS on CD4+ and CD8+ T-cells. In the presence of anti-CD28 (Biolegend clone: CD28.2; RRID:AB_314304), CD49d (Biolegend clone: 9F10; RRID:AB_2130040), and CD107 (Biolegend clone: H4A3; RRID:AB_1279055; APC) antibodies, PBMCs were stimulated for 6 hours with a DMSO negative control or 2 µg/mL of a high-quality (~95% purity) overlapping MARV GP peptide pool (15-mers overlapping by 11 amino acids; custom-made at GenScript, Piscataway, NJ). Brefeldin A protein transport inhibitor (Sigma cat: B6542) was added four hours before performing surface staining of CD3 (BD clone: SP34-2; RRID:AB_397044; Pacific Blue), CD4 (Tonbo clone: OKT4; RRID:AB_2621878; PerCP/Cy5.5) and CD8b (BD clone: 2ST8.5H7; RRID:AB_1645747; PE). DNase (Invitrogen cat: AM2224) and rhesus Fc receptor binding inhibitor (Thermo Fisher; RRID:AB_2572937) were added to reduce clumping and non-specific binding. Cells were subsequently washed in BD staining buffer and permeabilized using a Foxp3 / Transcription Factor Staining Buffer kit (Tonbo Biosciences cat: TNB-0607) as suggested by the manufacturer. After permeabilization, we stained for the intracellular markers Ki67 (BD clone: B56; RRID:AB_396302; FITC), IFN-gamma (Biolegend clone: B27; RRID:AB_2123321; PE/Cy7), and IL-2 (Biolegend clone: MQ1-17H12; RRID:AB_2562855; APC/Cy7).

For the regulatory T-cell (Treg) panel, we stimulated PBMCs overnight with media or 50 ng/mL of phorbol 12-myristate 13 acetate (PMA; Sigma cat: P8139) and 1 µg/mL of ionomycin (ION; Sigma cat: I0634) calcium salt from Streptomyces conglobatus. PBMCs were surface stained for the surface markers CD3 (BD clone: SP34-2; RRID:AB_397044; Pacific Blue), CD4 (Tonbo clone: OKT4; RRID:AB_2621878; PerCP/Cy5.5), and CD25 (Biolegend clone: M-A251; RRID:AB_2562489) for 30 minutes at 4 °C in the presence of DNase and rhesus Fc receptor binding inhibitor. We followed the instructions provided by the Foxp3 / Transcription Factor Staining Buffer Kit for permeabilization of PBMCs before adding intracellular fluorochrome-conjugated antibodies that target FOXP3 (Biolegend clone: 259D; RRID:AB_492982; PE), IL-10 (Biolegend clone: JES3-9D7; RRID:AB_2125385; PE/Cy7), and IL-4 (Biolegend clone: MP4-25D2; RRID:AB_315131; APC).

Approximately 200,000 events were collected on a FACS Canto II cytometer (BD Biosciences, San Jose, CA) for each panel using BD FACS Diva software and analyzed with FlowJo software (Tree Star, Ashland, OR). Live versus dead cells were distinguished by BV510 fixable viability dye (BD cat: 564406) for the ICS panels or by forward scatter (FSC) and side-scatter (SSC) properties for the monocyte and NK cell panels. Gating strategies are illustrated in Fig. S6. Compensation was calculated using BD CompBeads (BD; RRID:AB_1727537) and/or cells.

### Anti-MARV GP IgM and IgG ELISA

Sera collected at the indicated time points were tested for MARV GP-specific IgM and IgG antibodies by ELISA. MaxiSorp clear flat-bottom 96-well plates (44204 ThermoFisher, Rochester, NY) were coated overnight with 15 ng/well (0.15 mL) of recombinant MARV-Angola GP∆TM (∆TM: transmembrane region absent; Integrated Biotherapeutics, Gaithersburg, MD) in a sodium carbonate/bicarbonate solution (pH 9.6). Antigen-adsorbed wells were subsequently blocked with 4% bovine serum antigen (BSA) in 1 x PBS for at least two hours. Sera were initially diluted 1:100 and then two-fold through 1:12800 in ELISA diluent (1% BSA in 1 × PBS, and 0.2% Tween-20). After a one-hour incubation, cells were washed six times with wash buffer (1 x PBS with 0.2% Tween-20) and incubated for an hour with a 1:2500 dilution of horseradish peroxidase (HRP)-conjugated anti-rhesus IgM or IgG antibody (Fitzgerald Industries International, Acton, MA). RT SigmaFast O-phenylenediamine (OPD) substrate (Sigma; P9187) was added to the wells after six additional washes to develop the colorimetric reaction. The reaction was stopped with 3 M sulfuric acid 10–15 minutes after OPD addition and absorbance values were measured at a wavelength of 492 nm on a spectrophotometer (Molecular Devices Emax system, Sunnyvale, CA). Absorbance values were normalized by subtracting uncoated from antigen-coated wells at the corresponding serum dilution. End-point titers were defined as the reciprocal of the last adjusted serum dilution with a value ≥0.16.

### Plaque reduction neutralization test

Neutralization titers were calculated by determining the dilution of serum that reduced 50% of plaques (PRNT_50_). We incubated a standard 100 PFU amount of MARV with two-fold serial dilutions of serum samples for one hour. The virus-serum mixture was then used to inoculate Vero E6 cells for 60 minutes. Cells were overlaid with 2x EMEM agar medium, incubated for 6 days, and plaques were counted after 24 hours of 5% neutral red staining.

### Statistical analysis

GraphPad Prism (version 7.0) was used to conduct statistical analyses and prepare figures. To compare survival distributions and median time-to-death, log-rank tests were used. For the 23-plex cytokine analyses, a two-way ANOVA was used to detect differences between groups followed by a Bonferroni multiple comparisons test. Two-tailed t-tests were used for other statistical analyses.

## Supplementary information

Supplementary Materials.

## Data Availability

The RNA-seq datasets generated from this study are available in the NCBI Sequence Read Archive repository [accession number pending].
