# Supplementary material for: Immune correlates of postexposure vaccine protection against Marburg virus
Source: Sci Rep. 2020 Feb 20;10:3071. doi: 10.1038/s41598-020-59976-3 (PMC7033120; doi:10.1038/s41598-020-59976-3)
Supplement: Supplementary file 1 — Supplementary Materials. [file 41598_2020_59976_MOESM1_ESM.pdf]

Immune correlates of postexposure vaccine protection against Marburg virus

Authors: Courtney Woolsey[1,2], Allen Jankeel[3], Demetrius Matassov[4], Joan B. Geisbert[1,2], Krystle N. Agans[1,2], Viktoriya Borisevich[1,2], Robert W. Cross[1,2], Daniel J. Deer[1,2], Karla A. Fenton[1,2], Theresa E. Latham[4], Cheryl S. Gerardi[4], Chad E. Mire[1,2], John H. Eldridge[4,5], Ilhem Messaoudi[3], Thomas W. Geisbert[1,2]*

Affiliations:

[1]Department of Microbiology and Immunology, University of Texas Medical Branch, Galveston, TX 77555, USA.

[2]Galveston National Laboratory, University of Texas Medical Branch, Galveston, TX 77555, USA.

[3]Department of Molecular Biology and Biochemistry, School of Biological Sciences, University of California, Irvine, Irvine, CA 92697, USA.

[4]Department of Virology and Vaccine Vectors, Profectus BioSciences Inc., Pearl River, NY 10965, USA.

[5]Department of Immunology, Profectus BioSciences Inc., Pearl River, NY 10965, USA.

* Corresponding author: Thomas W. Geisbert, Ph.D., University of Texas Medical Branch, Galveston National Laboratory, 301 University Blvd, Galveston, Texas 77555-0610 (twgeisbe@utmb.edu).

Supplementary Materials

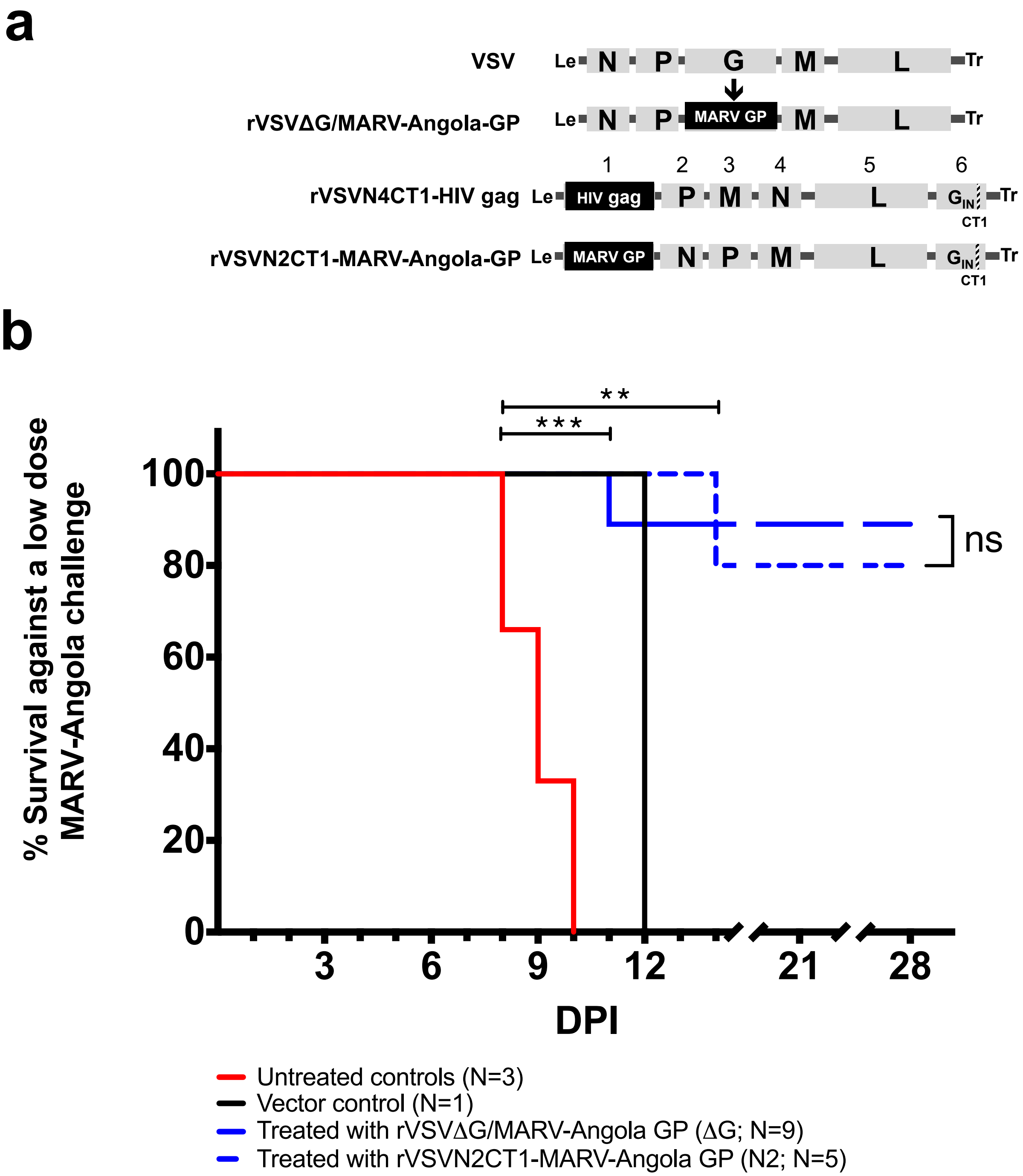

Fig. S1: rVSV vector design and survival of treated rhesus macaques.

a) rVSVΔG/MARV-Angola-GP (ΔG) was generated by swapping the native VSV Indiana glycoprotein gene with a MARV-Angola glycoprotein gene via restriction enzyme-mediated integration. To maximize antigen expression, the MARV-Angola-GP or HIV gag gene was cloned into the first genomic position of rVSVN2CT1-MARV-Angola-GP (N2) or the

rVSVN4CT1-HIVgag vector control. The N2 and control vectors were attenuated by shuffling the VSV nucleoprotein gene to the second or fourth position, respectively, and truncating the G cytoplasmic tail from 29 to 1 amino acid(s). The black and white-striped region within G denotes the amino acid substitution site. **b)** Kaplan-Meier survival curves of animals treated with rVSVΔG/MARV-Angola-GP (ΔG; solid blue line; N=9), rVSVN2CT1-MARV-Angola-GP (N2; segmented blue line; N=5), and rVSVN4CT1-HIV gag (vector control; black line; N=1). Groups treated with rVSV vectors expressing MARV-Angola-GP were significantly different than the untreated control group (red line; N=3). Statistical significance was not calculated against the vector control due to a lack of biological replicates. Abbreviations: VSV (Vesicular stomatitis virus); rVSV (recombinant vesicular stomatitis virus); N (VSV nucleoprotein); P (VSV phosphoprotein); M (VSV matrix protein); G (VSV glycoprotein); CT1 (truncated cytoplasmic tail); L (VSV polymerase); Le (leader); Tr (trailer); ΔG (the native VSV G is absent); MARV (Marburg virus, variant Angola); GP (MARV glycoprotein); N4 (the rVSV N is at position 4 in the genome); N2 (the rVSV N is at position 2 in the genome); HIV (human immunodeficiency virus); gag (group-specific antigen); PFU (plaque-forming units); DPI (days post-infection); ns (not statistically significant). Log-rank test ** $p \leq 0.01$, *** $p \leq 0.001$.

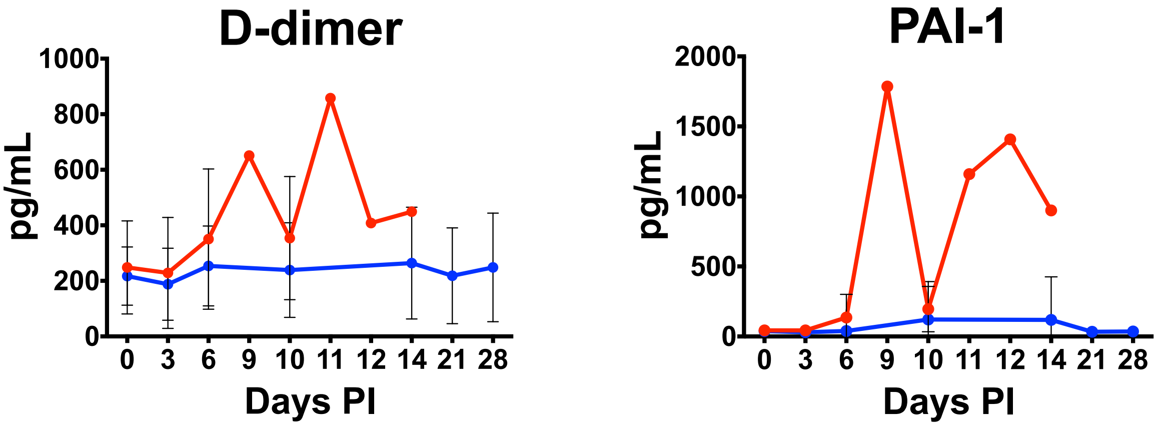

Figure S2. Plasma levels of coagulation-associated analytes in macaque subjects.
Red line represents the fatal dataset; blue line represents the treated survivor dataset. Abbreviations: PAI-1 (plasminogen activator inhibitor-1); PI (post-infection).

Animal	Day 0	Day 6	Day 10	Terminal or Day 28
Control 1	≤ 10	N.D.	N.D.	≤ 10
Control 2	≤ 10	N.D.	N.D.	≤ 10
Control 3	≤ 10	N.D.	≤ 10	≤ 10
Vector Control	≤ 10	N.D.	N.D.	≤ 10
ΔG Treated Fatal	≤ 10	≤ 10	≤ 10	≤ 10
ΔG Survivor 1	≤ 10	≤ 10	≤ 10	40
ΔG Survivor 2	≤ 10	≤ 10	≤ 10	40
ΔG Survivor 3	≤ 10	≤ 10	20	20
ΔG Survivor 4	≤ 10	≤ 10	≤ 10	40

ΔG Survivor 5	≤ 10	≤ 10	≤ 10	40
ΔG Survivor 6	≤ 10	≤ 10	≤ 10	40
ΔG Survivor 7	≤ 10	≤ 10	20	20
ΔG Survivor 8	≤ 10	≤ 10	≤ 10	40
N2 Fatal	≤ 10	≤ 10	≤ 10	≤ 10
N2 Survivor 1	≤ 10	≤ 10	20	40
N2 Survivor 2	≤ 10	≤ 10	≤ 10	20
N2 Survivor 3	≤ 10	≤ 10	20	40
N2 Survivor 4	≤ 10	≤ 10	≤ 10	40

Table S1: Neutralizing antibody titers.

Serum was evaluated for neutralizing antibody titers prior to challenge and terminally for untreated controls. Treated macaque sera were additionally evaluated on days 10 and 14 post-challenge. The reciprocal dilution titer of sera that neutralized ≥ 50% of viral plaques ($PRNT_{50}$ value) is reported. Abbreviations: ΔG, referring to individual monkey treated with rVSVΔG/MARV-Angola-GP; N2, referring to individual monkey treated with rVSVN2CT1-MARV-Angola GP; N.D., not determined.

Animal	Axillary LN	Inguinal LN	Liver	Spleen	Kidney	Adrenal Gland	Lung
Control 1	+	++	+++	++++	++	++	+++
Control 2	++++	++++	++++	++++	++++	++++	++++
Control 3	++++	++++	++++	++++	++++	++++	++++
Vector Control	++++	++++	++++	++++	++++	++	+++
ΔG Treated Fatal	+	++	+++	++++	++	++	+++
ΔG Survivor 1	N.D.	+	N.D.	+	N.D.	N.D.	+
ΔG Survivor 2	+	+	+	+	+	N.D.	N.D.
ΔG Survivor 3	+	N.D.	N.D.	N.D.	N.D.	N.D.	N.D.
ΔG Survivor 4	+	+	+	+	+	+	+
ΔG Survivor 5	+	+	+	+	+	+	+
ΔG Survivor 6	+	+	N.D.	+	+	+	+
ΔG Survivor 7	+	+	+	+	+	+	+
ΔG Survivor 8	+	+	+	+	+	+	+
N2 Fatal	+	++	++++	+++	++	++++	+++
N2 Survivor 1	+	+	+	+	+	+	+
N2 Survivor 2	+	+	+	+	+	+	+
N2 Survivor 3	N.D.	N.D.	+	+	+	+	N.D.
N2 Survivor 4	+	+	+	+	+	N.D.	+

Table S2: MARV viral load in tissues.

Samples from MARV-Angola-infected animals were normalized according to onset of viremia (mid-disease). Late-disease corresponded to 0-2 days before the animal succumbed, at peak

viremia. The median time points for these disease stages were used to define mid-disease (6 DPI) and late-disease (10 DPI) in the survivor dataset. +, < 8 logs; ++, 9 logs; +++, > 10 logs; ++++, > 11 LOGS. Abbreviations: N.D., not detected; LN, lymph node.

Animal	Group	Mid-Disease DPI	Late-Disease DPI
Control 1	Fatal	3	6
Control 2	Fatal	6	9
Control 3	Fatal	6	10
Vector Control	Fatal	6	10
ΔG Treated Fatal	Fatal	6	10
ΔG Survivor 1	Survivor	6	10
ΔG Survivor 2	Survivor	6	10
ΔG Survivor 3	Survivor	6	10
ΔG Survivor 4	Survivor	6	10
ΔG Survivor 5	Survivor	6	10
ΔG Survivor 6	Survivor	6	10
ΔG Survivor 7	Survivor	6	10
ΔG Survivor 8	Survivor	6	10
N2 Fatal	Fatal	10	14
N2 Survivor 1	Survivor	6	10
N2 Survivor 2	Survivor	6	10
N2 Survivor 3	Survivor	6	10
N2 Survivor 4	Survivor	6	10

Table S3: Normalization of samples for RNAseq, flow cytometry, and cytokine analyses. Samples from MARV-Angola-infected animals were normalized according to onset of viremia (mid-disease). Late-disease in the fatal group corresponded to 0-2 days before the animal died, at peak viremia. The median time points for these disease stages for the survivor group were used to define mid-disease (6 DPI) and late-disease (10 DPI).

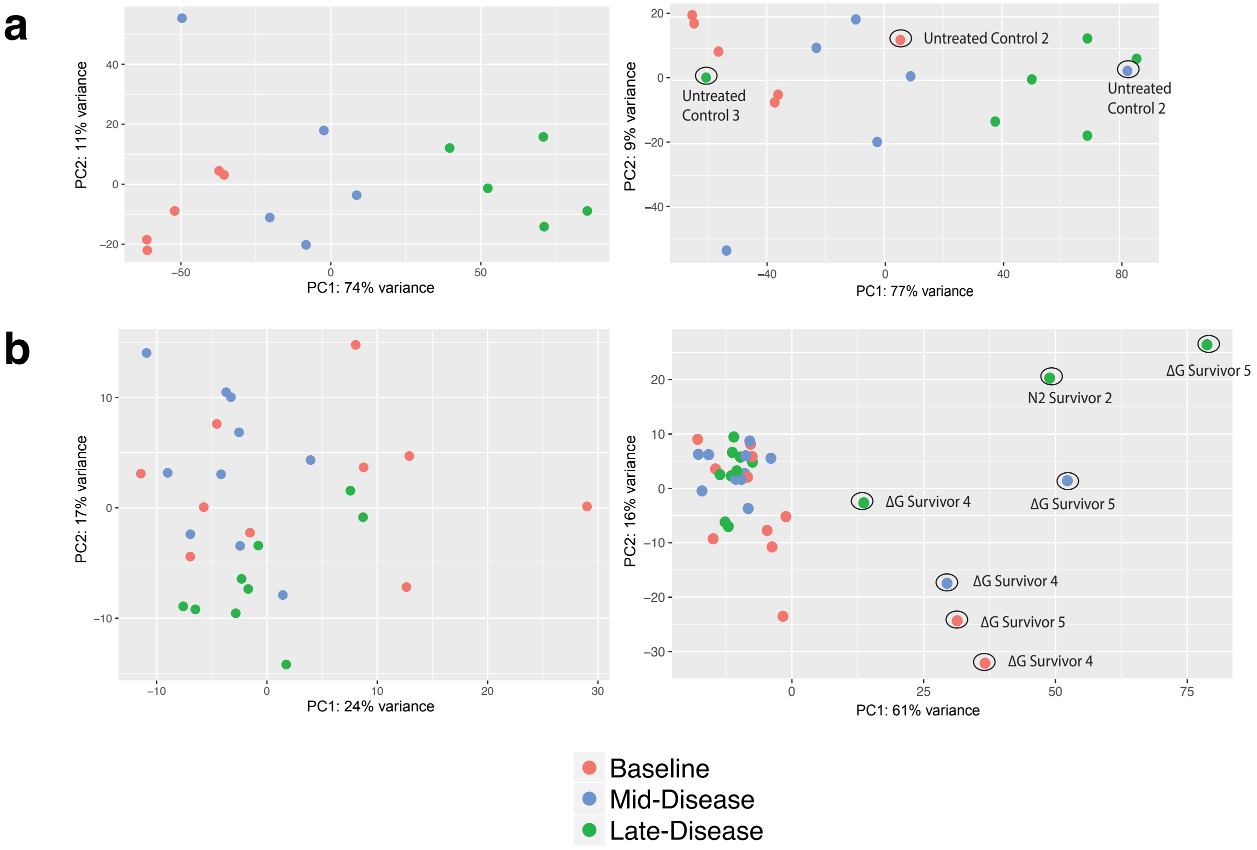

Figure S3: PCAs of survivor and fatal group transcriptomic datasets.

Principal component analyses (PCAs) of **a)** fatal and **b)** treated survivor samples with inclusion (right panels) or exclusion (left panels) of outliers (circled dots). No outliers were excluded from our transcriptomic analyses for either cohort as we determined that inclusion of these did not considerably skew these datasets. Baseline, mid-disease, and late-disease phases are represented by red, blue, and green dots, respectively.

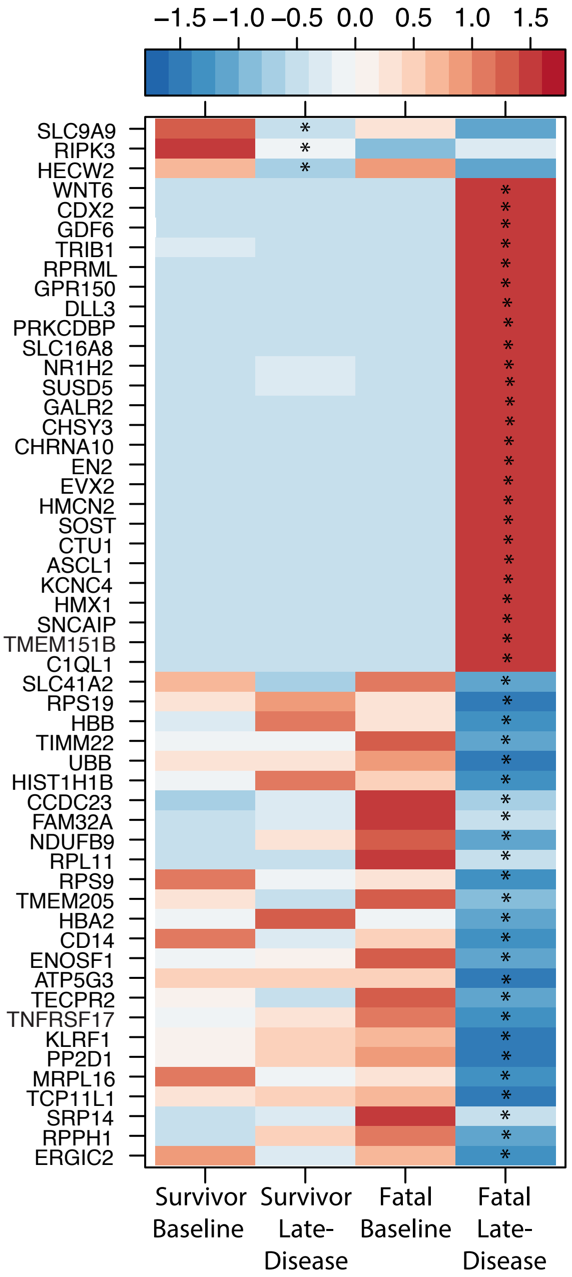

Fig. S4. Comparison of the most highly upregulated and downregulated DEGs at late-disease.

Heatmap of DEGs observed at late-disease. DEGs were calculated using EdgeR against a pre-challenge baseline to establish the most highly expressed genes. A scaled heatmap based on RPKM values within that set of genes (red represents increased expression while blue represents decreased expression); each column represents the median RPKM values for each time point. Only human homologs and protein-coding genes were analyzed. *: statistically significant, FDR-corrected p-value of ≤ 0.05.

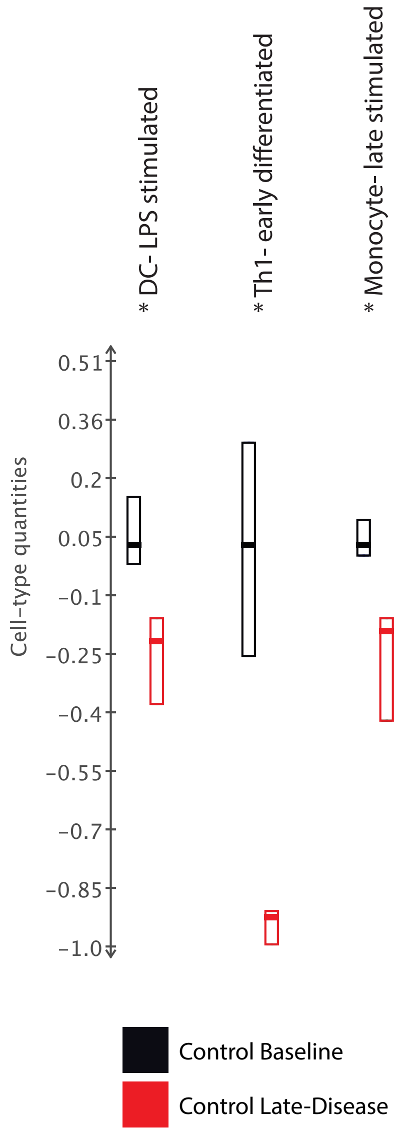

Figure S5. ImmQuant comparative view analysis of the relative contribution of immune cell subsets to differential gene expression within the control group.

ImmQuant uses a database based on genome-wide microarray expression profiling of human immune cells from reported studies. Results were calculated using the Digital Cell Quantifier (DCQ) algorithm with human-based FACS marker genes. The algorithm infers an increase or decrease in cell-type quantities relative to a Dy 0 baseline."*" indicates statistically significant putative changes in the cell subset frequency.

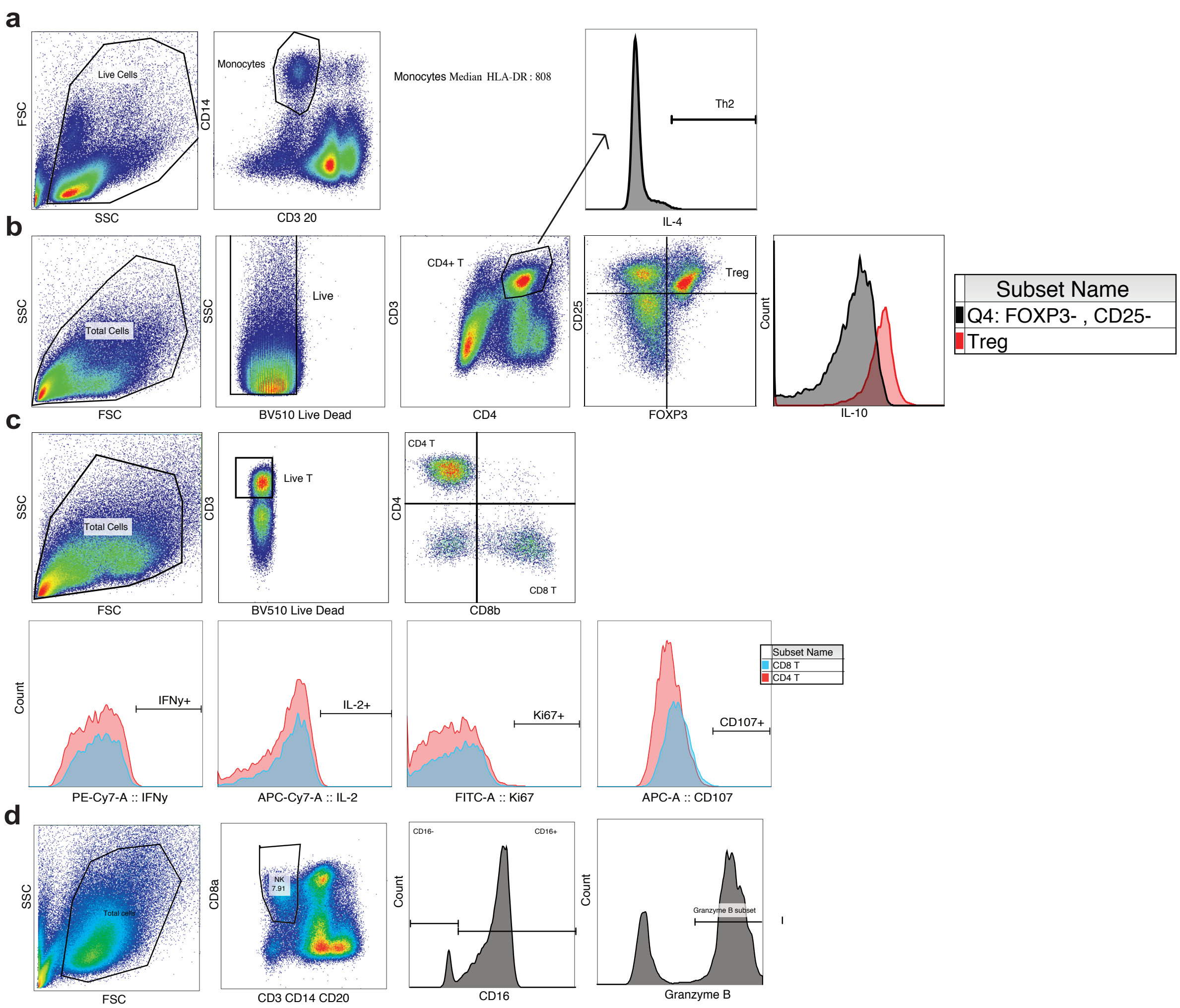

Figure S6. PBMC flow gating strategy.

a) Monocytes were identified based on lack of CD3 (T-cell) and CD20 (B-cell) expression, and positive CD14 expression. The mean fluorescence intensity of HLA-DR was then calculated within this population. **b)** After live/dead staining, regulatory T-cells (Tregs) were positively selected for the following markers: CD3, CD4, CD25, and FOXP3. We confirmed expression of IL-10 within the Treg subset. Th2 cells were gated on within the CD3+CD4+IL-4+ cell population. **c)** To identify Th1 and CTL populations, cells were stained with CD3, CD4, CD8b, IL-2, IFN-gamma, and CD107a fluorochrome-conjugated antibodies. T helper 1 (Th1) cells were identified by their expression of CD3, CD4, IL-2, and IFN-gamma. The frequency of proliferating Th1 cells was determined using the Ki67 marker. CTLs were identified by their expression of CD3, CD8, and the degranulation marker, CD107. **d)** NK cells were identified based on lack of CD3, CD20, and CD14 expression, and positive CD8α expression. Percentages of CD16+ and granzyme B+ populations were then determined within this population **a b c d)** Approximately 200,000 events were collected on a BD FACS Canto II cytometer and analyzed using FACS Diva and FlowJo software.
